# A Transcriptomic Atlas of Mouse Neocortical Layers

**DOI:** 10.1016/j.neuron.2011.06.039

**Published:** 2011-08-25

**Authors:** T. Grant Belgard, Ana C. Marques, Peter L. Oliver, Hatice Ozel Abaan, Tamara M. Sirey, Anna Hoerder-Suabedissen, Fernando García-Moreno, Zoltán Molnár, Elliott H. Margulies, Chris P. Ponting

**Affiliations:** 1 MRC Functional Genomics Unit, University of Oxford, Oxford OX1 3QX, UK; 2 Department of Physiology, Anatomy and Genetics, University of Oxford, Oxford OX1 3QX, UK; 3 Genome Technology Branch, National Human Genome Research Institute, National Institutes of Health, Bethesda, MD 20892-9400, USA

## Abstract

In the mammalian cortex, neurons and glia form a patterned structure across six layers whose complex cytoarchitectonic arrangement is likely to contribute to cognition. We sequenced transcriptomes from layers 1-6b of different areas (primary and secondary) of the adult (postnatal day 56) mouse somatosensory cortex to understand the transcriptional levels and functional repertoires of coding and noncoding loci for cells constituting these layers. A total of 5,835 protein-coding genes and 66 noncoding RNA loci are differentially expressed (“patterned”) across the layers, on the basis of a machine-learning model (naive Bayes) approach. Layers 2-6b are each associated with specific functional and disease annotations that provide insights into their biological roles. This new resource (http://genserv.anat.ox.ac.uk/layers) greatly extends currently available resources, such as the Allen Mouse Brain Atlas and microarray data sets, by providing quantitative expression levels, by being genome-wide, by including novel loci, and by identifying candidate alternatively spliced transcripts that are differentially expressed across layers.

## Highlights

► Online atlas of genome-wide transcription across neocortical layers ► Significant, replicated associations between disease genes and specific layers ► Widespread isoform switching across layers ► LincRNAs conserved, coexpressed across layers with neighboring protein-coding genes

## Introduction

Comparative and pathological studies suggest the mammalian cerebral cortex to be the anatomical substrate of higher cognitive functions including language, episodic memory, and voluntary movement (Jones and Rakic, 2010; Kaas, 2008; Rakic, 2009). The cerebral cortex has a uniform laminar structure that historically has been divided into six layers (Brodmann, 1909). The upper layers (1 to 4) form localized intracortical connections (Gilbert and Wiesel, 1979; Toyama et al., 1974) and are thought to process information locally. The deep layers of the cortex, 5 and 6, form longer-distance projections to subcortical targets (including the thalamus, striatum, basal pons, tectum, and spinal cord) and to the opposite hemisphere. Some layer 5 neurons are among the largest cells of the brain and exhibit the longest connections. Layer 6b in mouse neocortex is a distinct sublamina with characteristic connections, gene expression patterns, and physiological properties (Hoerder-Suabedissen et al., 2009; Kanold and Luhmann, 2010).

Understanding how neurons and glia are organized into layers to assemble into functional microcircuits (Douglas and Martin, 2004) is one of the first steps that will be required to relate anatomical structures to cellular functions. Subclasses of pyramidal neurons and interneurons populate specific layers, each characterized by a different depth in the cortex with a specific pattern of dendritic and axonal connectivity (Jones, 2000; Lorente de No, 1949; Peters and Yilmaz, 1993). However, analyzing these laminar differences is difficult and often suffers from subjectivity (Zilles and Amunts, 2010).

The currently available repertoire of markers that allow the distinction of cortical layers and of many neuronal and glial subtypes is rapidly improving because of developments in cell sorting and gene expression analysis (Doyle et al., 2008; Heintz, 2004; Miller et al., 2010; Molyneaux et al., 2007; Monyer and Markram, 2004; Nelson et al., 2006; Thomson and Bannister, 2003; Winden et al., 2009). These molecular tags allow highly specific classes of neurons and glia to be monitored, modulated, or eliminated, thereby providing greater insights into cortical neurogenesis and the classification of lamina specific subclasses of cells.

Laminar molecular markers were first identified by studying single protein-coding genes (Hevner et al., 2006; Molyneaux et al., 2007; Yoneshima et al., 2006) but more recently, high-throughput in situ hybridization (Hawrylycz et al., 2010; Lein et al., 2007; Ng et al., 2010) and microarrays (Oeschger et al., 2011; Arlotta et al., 2005; Hoerder-Suabedissen et al., 2009) have enhanced our understanding of mammalian cortical expression patterns for thousands of genes. However, these probe-based approaches are necessarily tied to existing gene models and exclude thousands of noncoding loci and most alternative splice variants. Despite these limitations and the quality-control challenges posed by industrialized automated histology platforms, the Allen Mouse Brain Atlas is a superb resource for qualitative information on mouse brain gene expression. Although microarrays are more quantitative than in situ hybridizations, they exhibit a narrow dynamic range compared to the six orders of magnitude that are easily spanned by RNA-seq data (Bradford et al., 2010; Trapnell et al., 2010). Instead, we sought to provide accurate and comprehensive genome-wide profiles of transcript and gene expression across cortical cell layers by deep sequencing of both coding and noncoding polyadenylated transcripts across adult mouse neocortical layer samples. Owing to cells traversing layer boundaries and dissection limitations, we constructed naive Bayes classifiers that inferred patterns of layer-specific expression.

## Results

### Gene Expression Levels across Dissected Samples Reflect Known Neocortical Layers

**Figure 1:**
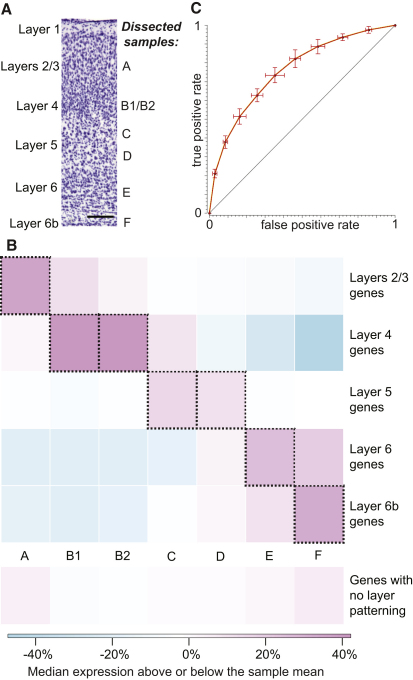
Laminar Dissections Correspond to Known Cytoarchitectural Layers (A) Samples were dissected manually using microsurgical scalpel under binocular microscope from coronal sections of primary somatosensory cortex. Nissl stain of RNA reveals layers with different cell densities across adjacent layers 1–6b and their correspondence to laminarly dissected samples (scale bar represents 200 μm). (B) Genome-wide RNA-Seq gene expression patterns (see also Table S1) across samples A–F recapitulate gene expression preferences in layers 2–6b known from manual curation (Lein et al., 2007). The heatmap reflects the relative RNA-Seq expression across samples of “patterned” genes known to be expressed in one (or more) neocortical layer. Demonstrated correspondences between dissected layers and samples are highlighted with outlined red panels on the descending diagonal. The lowest set of panels indicates genes that show no layer patterning. If genes *g_l_* are annotated (Lein et al., 2007) as being preferentially expressed in layer *l* and *f_sgl_* is the fractional expression of gene *g_l_* in sample *s* relative to expression in all samples, then *t_sl_* is the median of (*f_sgl_*) over genes *g_l_*. The heat map represents [*t_sl_* – mean_all layers_*_l_* (*t_sl_*)]/[mean_all layers_*_l_* (*t_sl_*)]. (C) This correspondence allowed apparent layer enrichment to be predicted from expression across dissected samples with naive Bayes classifiers. The receiver operating characteristic (ROC) curve for the layer 5 classifier is shown, as it yields a middling *a*rea *u*nder the ROC *c*urve (AUC) (Table 1; Figure S1). Error bars indicate sample standard deviations based on 10-fold cross validation.

Polyadenylated RNA was extracted from dissections of neocortical layers from eight male, 56-day-old, C57BL/6J mice. RNA samples A-F were derived from six adjacent laminar segments (from superficial to deep layers, respectively) of mouse primary somatosensory cortex (S1) from two sets of four littermates dissected under a binocular microscope with a microsurgical scalpel (Figure 1A). Samples B1 and B2 represent biological replicates of the second segment from four littermates each. Subsequently, at least 50 million paired-end cDNA fragments passing Illumina's quality filter were deep sequenced from each dissected sample on the Illumina GA IIx platform, thereby providing a comprehensive genome-wide view of transcription. Sequenced cDNA fragments spanned 25% of the mouse genome and overlapped 18,960 protein-coding genes (83%) with 16,340 protein-coding genes (72%) expressed above a level of 0.1 *f*ragments per *k*ilobase of *e*xon model per *m*illion reads mapped (FPKM) (Mortazavi et al., 2008; Trapnell et al., 2010) (Table S1). Half of all transcripts derived from just 2% of expressed genes and the most highly expressed genes were generally of mitochondrial origin (see also Belgard et al., 2011). 10% of mouse genome sequence located outside of known protein-coding, pseudogene, tRNA, rRNA, and short RNA gene loci, was expressed in at least one sample, including 1,055 long intergenic noncoding (lincRNA) loci (Table S1; Belgard et al., 2011) (Ponting et al., 2009).

Because of layer curvature, cells crossing multiple layers, and dissection limitations, it was not expected that samples should correspond precisely to individual layers. Indeed, although Spearman's rank correlation coefficient for expression levels between B1 and B2 was high at 0.965, overlapping dissections resulted in several higher interpair correlations (see also Belgard et al., 2011). Nevertheless, known marker genes (Lein et al., 2007) for layers 2/3, 4, 5, 6, and 6b demonstrated high concordance between individual samples and specific layers (Figure 1B, Belgard et al., 2011).

We compared our RNA-seq results with those previously obtained using microarrays for layer 6 and 6b from anterior cortex (putative S1) of postnatal day 8 mice (Hoerder-Suabedissen et al., 2009). RNA-seq levels for samples E and F were highly and significantly concordant with microarray expression levels for layers 6 and 6b despite methodological differences and the difference in age (Supplemental Experimental Procedures): 85% (147 of 173) of genes whose expression was found, with microarrays, to be significantly lower in layer 6 versus 6b also showed lower expression in sample E versus F; significant concordance was also found for 87% (385 of 441) of genes significantly lower in layer 6b versus 6, compared with sample F versus E (each test, p < 2 × 10^−16^, two-tailed binomial test relative to a probability of 0.5).

### Thousands of Genes Are Patterned across Neocortical Layers

**Figure 2:**
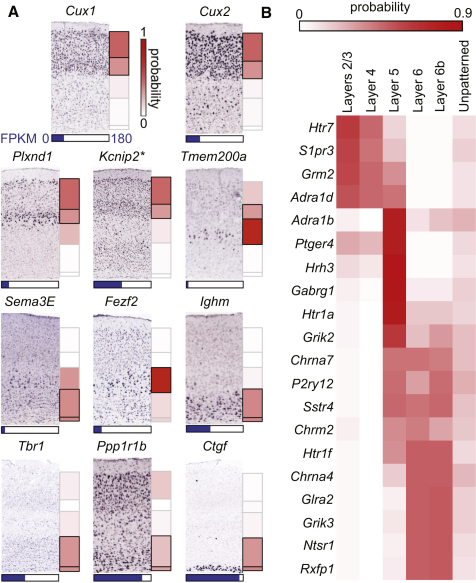
Classifiers Recapitulate Classical Layer Markers, Enrich Existing In Situ Hybridizations with Quantitative Data, and Greatly Expand the Number of Known Genic Layer Markers (A) Classifier predictions typically recapitulated known layer markers. Calibrated layer enrichment probabilities for eleven classical layer markers (Molyneaux et al., 2007) are represented to the right of the corresponding in situ hybridization image reproduced with permission from the Allen Mouse Brain Atlas (Lein et al., 2007) (Supplemental Experimental Procedures). Boxes outlined in black indicate that the classifier predicted the gene to be “enriched” in that layer. Blue bars beneath each image represent the highest expression level of that gene in any sample, highlighting an additional dimension of information. Common synonyms for these genes include *Darpp32* (*Ppp1r1b*), *Igh6 (Ighm*), and *C030003D03Rik* (*Tmem200a*). ^∗^*Kcnip2* was the only image from a sagittal, as opposed to a coronal, section. (B) In addition to increasing the number of known (Lein et al., 2007) layer-patterned genes by 3- to 4-fold (Figure S3), these classifiers are also informative for genes that already have high-throughput in situ hybridization images. This is illustrated by calibrated layer enrichment probabilities of 20 of the most highly patterned genes encoding receptors, selected blindly to status in the Allen Mouse Brain Atlas, in which 9 were qualitatively consistent, 4 had a faint signal, 3 had a ubiquitous signal, 3 were qualitatively inconsistent, and 1 had a failed probe (Table S3). Our classifiers provide an objective evaluation of cortical patterning for these genes with ambiguous images.

We next predicted 6,734 “patterned” genes that are preferentially expressed in one or more layers and 5,689 “unpatterned” genes that were expressed more uniformly across all layers. For this, layer expression for 2,200 genes annotated from in situ hybridization images (see also Belgard et al., 2011) was used for training a naive Bayes classifier for each layer 2–6b. (Annotated marker genes were insufficient to permit training of a reliable classifier for layer 1.) These curations are generally consistent with the literature and other expression data sets (Allen Institute for Brain Science, Top 1,000 Genes Analysis: Validation of Frequently Accessed Genes in the Allen Mouse Brain Atlas, http://mouse.brain-map.org/pdf/Top1000GenesAnalysis.pdf, 2010). A classifier was also constructed to separate patterned from unpatterned genes. Classifier generalization accuracies were assessed with 10-fold cross-validation (Figure 1C; Table 1; Figure S1), and smoothed calibration curves were constructed for the resulting predicted probabilities to arrive at accurate estimates of enrichment likelihood (Figure S2). Finally, these classifiers were applied to both known and previously unknown genes and transcripts (Table S2; Belgard et al., 2011). A total of 11,410 known genes (10,261 protein-coding) were expressed at sufficiently high levels for their layer patterning to be classifiable. Predicted layer expression patterns typically recapitulated both the literature (Figure 2A) and the results of the high-throughput curation-based approach (Table 1).

Upon correcting for false positives and false negatives, we found that an estimated 5,835 of these 11,410 classifiable known genes (51%) were expressed preferentially in one or more layers (Table 1, Supplemental Experimental Procedures). Among protein-coding genes, 4,464 (44%) had a higher calibrated probability of being enriched in at least one layer than of being unpatterned. Transcripts from 66 lincRNA loci (see below) were classified as being patterned (Table S7).

Of 291 genes encoding receptors and ion channels, 108 were expressed highly enough to be classified and 82 of these were predicted to be patterned across the layers (Table S3). Layer enrichment probabilities of the 20 most highly patterned receptors are shown in Figure 2B and are generally consistent with previous observations (Table S3).

Some of this patterning reflected known cell types. Neuron-enriched genes (≥1.5-fold; Cahoy et al., 2008) were 63% more likely to be patterned than unpatterned (p < 0.0001; two-tailed Chi-square test with Yates correction). There were no significant differences among the layers in their proportions of neuron-enriched genes, suggesting these differences in neuron-enriched genes may reflect laminar diversity of neuronal subtypes rather than differences in relative populations of neurons in aggregate. In contrast, astrocyte-enriched genes (≥1.5-fold; Cahoy et al., 2008) were 17% less likely to be patterned than unpatterned (p = 0.0007; two-tailed Chi-square test with Yates correction). Oligodendrocyte-enriched genes (Cahoy et al., 2008) were found almost exclusively in the deepest samples (see Belgard et al., 2011), matching previous observations that oligodendrocytes are rare in the neocortex except in the deepest layers (Tan et al., 2009). Likewise, the gene encoding the specific and robust microglia marker F4/80 (Cucchiarini et al., 2003; Perry et al., 1985) monotonically increased in expression with samples derived from deeper layers.

### Isoform Switching across Layers

**Figure 3:**
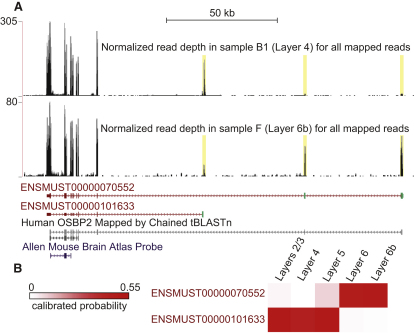
*Osbp2* Shows Signs of Differential Expression of its Isoforms across Layers (A) Normalized read depth across samples A–F in the mouse chr11:3,593,238-3,771,939 region. Two isoforms account for most expression from this locus: ENSMUST00000070552 and ENSMUST00000101633. Yellow boxes highlight read coverage in mutually exclusive exons outlined in green. The FPKM of ENSMUST00000070552 was significantly higher in sample F than in sample B1, whereas the FPKM of ENSMUST00000101633 was significantly higher in sample B1 than in sample F, indicating that both transcripts are differential expressed across samples. The probe used for in situ hybridization in the Allen Mouse Brain Atlas (RP_050125_02_B09) does not discriminate between these isoforms. (B) Classifiers predict that ENSMUST00000070552 is enriched in deeper layers whereas ENSMUST00000101633 is enriched in upper layers. See Figure S4 for a complex neurologically relevant example and Table S4 for the layer enrichment probabilities of additional candidate alternatively spliced genes.

**Figure 4:**
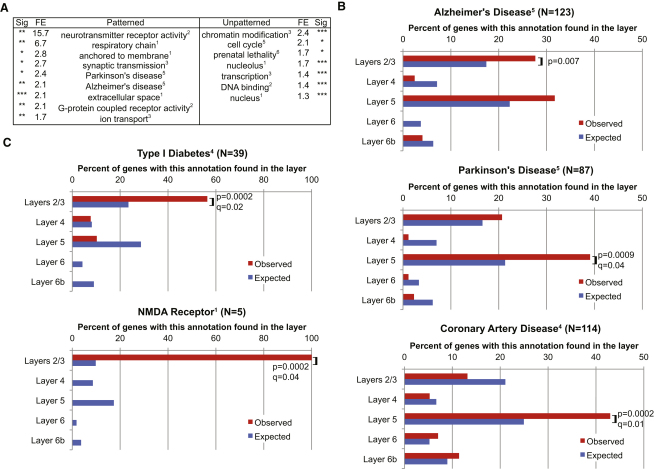
Genes Enriched in Expression for Specific Layers Are Significantly Associated with Specific Diseases and Functions (A) Selected functional classes of genes whose expression across neocortical layers is significantly patterned (all genes predicted to be preferentially expressed in one or more layers) or unpatterned (predicted to have no layer enrichment). Significance was assessed with a two-tailed Fisher's exact test, followed by a q value multiple testing correction. All of these terms were replicated with a p value of lower than 0.05 in either dorsal or lateral cortex (Table S6). The full list of significant terms is provided in Belgard et al. (2011). Sig, significance (^∗^q < 0.05; ^∗∗^q < 0.01, ^∗∗∗^q < 0.001); FE, fold enrichment over expected. (B) Distributions of genes across layers of selected functions that were significantly patterned in either the original set or one of the replication sets with a Bonferroni-corrected p < 0.05. See Table S6 for a complete nonredundant list of enriched terms and also for both replications. Where the observed percentage of genes in a layer differs significantly from the expected percentage of genes in a layer, we list the appropriate p or q values smaller than 0.05, as assessed by a bias-corrected simulation method (Supplemental Experimental Procedures). (C) Distributions of genes across layers of selected functions that were significantly enriched in one layer (q < 0.05), as assessed by a bias-corrected simulation method (Supplemental Experimental Procedures). Terms were drawn from the following databases, as cited: ^1^Gene Ontology (Ashburner et al., 2000) Cellular Component, ^2^Molecular Function, and ^3^Biological Process; ^4^1:1 mouse orthologs of human genes previously associated with human traits and diseases (Chen et al., 2010); ^5^Kyoto Encyclopedia of Genes and Genomes (Kanehisa et al., 2004); and ^6^mouse knockout phenotypes (Blake et al., 2011). See also Figure S5 and Table S5.

Two thousand three genes had at least two transcript isoforms that were each classifiable. One thousand six hundred forty-six of these genes (82%) showed differential patterns of alternative splicing across sequenced samples (Table S5). Seven hundred nineteen genes, including eight encoding receptors, additionally had divergent predicted patterns of layer enrichment (Figure 3; Table S4). The differential splicing across layers of *Mtap4*, the most connected hub gene in Alzheimer's disease (Ray et al., 2008), is one example of the potential neurological relevance of this set (Figure S4). *Mtap4* encodes isoforms of MAP4 with differing microtubule-stabilization properties (Hasan et al., 2006) that have been proposed to regulate the dynamic behaviors of extending neurites (Hasan et al., 2006), structures lost or altered in the earliest stages of Alzheimer's disease (Knobloch and Mansuy, 2008). Most Alzheimer's disease genes were enriched in either layers 2/3 or layer 5 (Figure 4B; Table S6), which were dominated by an isoform having an additional tau domain compared to the isoform that dominated layers 6 and 6b (Figure S4). We found that in situ hybridization was sometimes unable to detect minor isoforms in the cortex that were clearly detectable by RNA-seq, which once more underscores the greater dynamic range of transcriptome sequencing.

The set of genes with differentially expressed alternative transcripts also reflected the action of splicing factors. For example, the splicing factor Fox-2, encoded by *Rbfox2*, is known to be sufficient for exon inclusion in *Ewsr1* in a dose-dependent manner (Underwood et al., 2005), producing a brain-enriched isoform (Melot et al., 2001). Consistent with this, we found the FPKM of *Rbfox2* across samples was strongly correlated with both the isoform fraction (*r* = 0.85; p < 0.01, one-tailed) and the FPKM of the brain-enriched *Ewsr1* isoform (ENSMUST00000102930; *r* = 0.87; p < 0.01, one-tailed). In contrast, the total FPKM of *Ewsr1* was significantly correlated neither with the isoform fraction (*r* = −0.47; not significant) nor with the FPKM of this brain-enriched isoform (*r* = 0.19; not significant).

### Biological Associations for Genes Expressed Highly in Specific Layers

Genes that exhibited patterned expression were, in general, very different from those expressed more evenly across the cortical layers. They were more likely to encode specific receptor types and proteins involved in synaptic transmission and ion transport (Figure 4A; Belgard et al., 2011). By contrast, unpatterned genes tended to possess housekeeping cellular roles that less often contribute to cell type-specific functions. An exception to this housekeeping generalization is mitochondrial genes, which were significantly and strongly enriched in layer 5 (Table S6). We note that the extremely large layer 5 pyramidal neurons, which are the only cell types that extend axons beyond the skull, are known to have high energetic demands (Guillemot et al., 2006; Molnár and Cheung, 2006; Molyneaux et al., 2007). Unpatterned genes appear to be more important in early development: they were significantly more likely to result in prenatal lethality when disrupted in mice (Figure 4A).

Cells in cortical layers are proposed to have differing biological roles. We sought transcriptomic evidence for these roles by identifying, for each layer in turn, molecular annotations of genes that were more abundant than expected from a bias-corrected random sampling of all 11,410 classifiable genes (Supplemental Experimental Procedures; Belgard et al., 2011). Molecular annotations were drawn from a variety of sources, including the Gene Ontology (Ashburner et al., 2000), genomic intervals associated with human diseases and traits identified by genome-wide association studies (Chen et al., 2010), and mouse knockouts (Blake et al., 2011), and only results retained after application of a 5% false discovery rate threshold for each set are reported.

Expression of genes encoding subunits of the NMDA receptor were enriched in layers 2/3: expression of all five classifiable genes were identified as being significantly concentrated in these layers, a number much higher than expected simply by chance (Figure 4C). NMDA receptor subunits are expressed across the cortex and, despite being largely responsible for physiological responses of cells in layers 5 and 6, they are known to be preferentially expressed in layers 2/3 (Conti et al., 1997; Currie et al., 1994). In subsequent replications (see below), a majority of layer-enriched NMDA receptor subunit genes were enriched in layers 2/3 (Table S6). Genes encoding proteins localized to the extracellular space or region were expressed at significantly higher levels in layers 2/3, 4, 6, or 6b (Table S6). Surface markers in brain cells are often involved in various signaling processes, from guidance to synapse formation (Maruyama et al., 2008; Uziel et al., 2006; Yamamoto et al., 2007).

We identified significant association of layer-enriched expression for genes whose human orthologs lie within genomic intervals previously associated with disease. In particular, mouse orthologs of human type 1 diabetes- and rheumatoid arthritis-associated genes were unusually abundant among layers 2/3-enriched genes (Figure 4C, Table S6). These findings reflected nearly all of these genes' locations being within the major histocompatibility complex (MHC) region. Indeed, genes in the MHC region were 34% more likely than randomly selected genes (p < 10^−6^; case resampling bootstrap) to have enriched expression in layers 2/3. Many of these examples are confirmed by in situ hybridizations in the Allen Mouse Brain Atlas (Figure S5). It was the nonimmune genes of the MHC region whose expression was particularly enriched in layers 2/3 (Figure S5) and that contributed to the significant associations observed with these two diseases. In subsequent replications, a majority of layer-specific MHC genes and a large minority of all MHC genes were again enriched in layers 2/3 (Table S6).

Another apparent disease association links mouse genes preferentially expressed in layer 5 with human genes in the Parkinson's disease pathway (Figure 4B). This, however, is likely to reflect the involvement of mitochondrial dysfunction in Parkinson's disease (Abou-Sleiman et al., 2006; Burbulla et al., 2010) and the prominent expression of mitochondrial and metabolic genes in this layer (Table S5; Table S6). For example, *Lrrk2*, whose human ortholog is mutated in familial Parkinson's disease (Abou-Sleiman et al., 2006), is expressed prominently in rodent neocortical layers 2/3 and 5 (this study; Lein et al., 2007), specifically pyramidal neurons, and is associated with mitochondrial markers (Biskup et al., 2006). In subsequent replications, nearly half of Parkinson's disease-related genes were enriched in layers 2/3 and nearly half in layer 5 (Table S6). Twenty-nine of the thirty-six genes with known enrichments from in situ hybridization were manually curated as enriched in layer 5 (Table S6).

### Gene Sets and Functional Differences Replicated in the Dorsal Cortex and Lateral Cortex

We subsequently replicated these gene sets and functional differences in both dorsal cortex (including, but not limited to, S1) and lateral cortex (not overlapping S1; including, but not limited to, S2) in experiments involving different mice, a different dissecting anatomist, different technicians making the cDNA, sequencing libraries with a different protocol, updated sequencing chemistry and software, different filtering criteria, and additional variables used to train the classifiers (Supplemental Experimental Procedures). The overall concordance rates for genes of the two replication predictions with the original predictions ranged from 64%–96%, and a majority of genes predicted to be enriched in one experiment were also predicted to be enriched in the other (Table 2). All but one of the 39 significant functional differences in Table S6 were changed in the same direction in both replication sets, and 87% of these, including all disease associations, also had a replicated p value smaller than 0.05 in either dorsal or lateral cortex (Table S6). Most, but not all, genes were similar in patterning between dorsal and lateral cortex (Figure S6), confirming previous observations (Hawrylycz et al., 2010).

### LincRNA Expression Varies across Neocortical Lamination

We next turned to the 1,055 lincRNA loci, expressing 1,879 multiexonic transcripts (see also Belgard et al., 2011), which we identified as being expressed in the cortical cell layers, usually at low levels. A large majority (67%) of these loci were novel, in that they had no overlap with annotated noncoding RNA loci (Rhead et al., 2010), likely because of their low expression. Sequence constraint, an indicator of functionality, for these loci was similar to that seen for other lincRNA locus sets (Marques and Ponting, 2009) (Figure S7; Belgard et al., 2011). These lincRNA loci also exhibited experimental hallmarks of transcribed loci; they significantly overlapped DNase I hypersensitivity sites and histone methylation marks that are associated with active transcription in neuronal precursor cells (Figure S7).

Some lincRNA loci have been predicted (Ponjavic et al., 2009), and experimentally verified (Ørom et al., 2010), to act in *cis* by regulating the expression of genomically proximal protein-coding genes. Some lincRNAs expressed in our cortical samples may also possess such functions, particularly in the regulation of patterns of expression across cortical layers. This is supported by patterned protein-coding gene loci being 39% more likely to be adjacent to one or more of these lincRNA loci than expected by chance (p < 10^−4^), and lincRNA expression across cortical layers being more often correlated, positively or negatively, with expression of their protein-coding genomic neighbors if these genes were patterned than if they were unpatterned (p < 0.02; see also Supplemental Experimental Procedures and Belgard et al., 2011). Furthermore, we found a significant enrichment of cortical lincRNA transcription across enhancers defined in in vitro neuronal culture (Kim et al., 2010) (2-fold increase, p < 10^−4^). Finally, because Evofold-predicted (Pedersen et al., 2006) RNA structures were substantially enriched in these transcripts (2-fold increase, p = 2 × 10^−3^), the activity of some cortical lincRNAs is likely to be mediated by their secondary structures.

**Figure 5:**
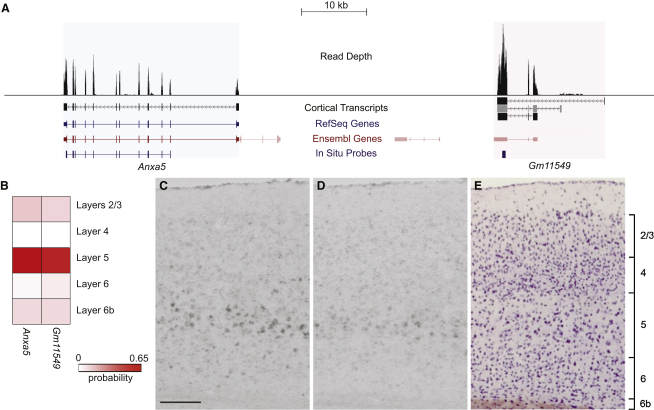
*Anxa5* Cortical Expression Overlaps with that of its Neighboring Cortical lincRNA (*Gm11549*) (A) Read depth from all cortical RNA-seq data, and multiexonic de novo transcripts, in the mouse chr3:36,347,839-36,431,074 region. Of all predicted Ensembl [coding (red) and noncoding (pink)] and RefSeq gene annotations in this region, only *Anxa5* and *Gm11549* were expressed. All transcripts overlapping *Gm11549* were predicted with high confidence to be noncoding except for the one marked in gray, which had a short open reading frame covering less than 8% of the mostly repeat-derived transcript. Locations of the in situ probes are indicated in purple. (B) Calibrated cortical layer enrichment probabilities for *Anxa5* (ENSMUSG00000027712) and *Gm11549* (de novo model XLOC_901616). Both genes were predicted to be enriched in layer 5 (uncalibrated probability > 0.5). (C and D) In situ hybridizations for (C) *Anxa5* and (D) *Gm11549* confirmed that their expression in the mouse cortex was as predicted. (E) Cortex stained for Nissl. The maximum read depth at any position in this region, combining all samples, was 2,451. The scale bar represents 200 μm. See Figure S7 for further lincRNA properties and Table S7 for layer enrichments of patterned lincRNA transcripts.

At least 76 of these cortical lincRNAs, from 66 loci, exhibited differential expression across cortical layers. Importantly, patterned lincRNAs were 63% more likely to be adjacent to patterned protein-coding genes than expected by chance (p < 0.01) (Figure 5), supporting a role of patterned lincRNAs in the regulation of cortical genetic architecture. As illustrated in Figure 5 and Figure S7, patterned lincRNAs transcripts are bona fide layer specific markers. At least one lincRNA was more highly expressed outside of cortex (subventricular zone, dentate gyrus, and Purkinje cells of the cerebellum; Figure S7), suggesting that such lincRNAs will be of broader interest to neuroscientists.

## Discussion

We have demonstrated differential expression of protein coding or noncoding alternative transcripts and genes across expression levels spanning over six orders of magnitude for individual layers of the mouse neocortex with what is, to our knowledge, the first deep sequencing of transcriptomes from separate mammalian neocortical layers. An interactive interface to explore the data, and links to download them, are available from Belgard et al. (2011). This resource should assist future studies that seek a detailed molecular and functional taxonomy of cortical layers and neuronal cell types. Our results increase by 3 to 4-fold the number of known (Lein et al., 2007) layer-specific marker genes (Figure S3) and furthermore introduce 66 lincRNA loci as new markers. These markers can assist studies of cortical cell types, neurodevelopment, and comparative neuroanatomy. Our data even augment known marker genes by providing a more objective grounding for their laminar classifications on the basis of quantitative expression level. They also reveal novel observations on each layer's neurological functions that lead to new lines of enquiry, for example regarding the roles of Alzheimer's disease genes or MHC genes in layers 2/3 or of mitochondrial biology in layer 5. Our findings in mouse are expected to be highly relevant to human biology owing to these species' strong similarities in brain transcriptomes (Strand et al., 2007) and to the similarities of layer markers between mouse and ferret (Rowell et al., 2010), which is an evolutionary outgroup to rodents and primates. Even unexpected findings, such as the significant and replicated association between coronary artery disease and layer 5 expression, may reflect genetic underpinnings of previously described clinical associations between vascular and neurological disease (Beeri et al., 2006; Santos et al., 2009).

Our application of a machine learning classifier to carefully annotated high-throughput in situ hybridizations (Lein et al., 2007) yields expression levels and predictions of laminar patterning that are based on transcripts, as well as on genes, and on noncoding loci, as well as on known genes. The expression levels assessed by RNA-seq are more sensitive to smaller differences, and these can be explored on Belgard et al. (2011). Results reflect the genome as a whole (except for repetitive sequence within which mapping of reads is problematic), rather than for the limited sequence targeted by microarray probe sets. This revealed numerous lincRNA transcripts, mostly novel, which were evolutionarily constrained, sometimes imprinted (Gregg et al., 2010), and at least one that was most strongly expressed outside of cortex, opening new avenues for research into their extracortical functions. Additionally, we found transcripts from the same gene exhibiting expression divergence across neocortical layers, which should be investigated for potential physiological consequences. None of this would have been possible with currently available microarray-based methods.

Nevertheless, our approach will be limited by imperfections in dissection, and by contributions to one layer of transcripts emanating from radial processes of cells whose soma lie in another. These limitations will degrade the classifier's performance and hence will contribute to a large number of genes (56%) whose maximum predicted probabilities lie below 0.5. Nevertheless, the approach still provides at least a 10-fold difference in the relative probability of enrichment in different layers for over 10,857 (95%) classifiable genes and thus is effective at inferring transcriptional levels among mixed populations of cells in their *milieu*, rather than for cells that have been sorted, purified, or microdissected (Markram et al., 2004; Molyneaux et al., 2007; Nelson et al., 2006). Indeed, there is a recent demand for integration of neuronal, glial, and vascular interactions on a molecular and cellular level within the same neuronal structures (Neuwelt et al., 2011). Our findings make possible future comparisons of whole transcriptomes across both isolated cell-types and cell layers that should yield further insights into the molecular components of the neuronal circuitry underlying higher brain functions. Finally, the data set shall enable us to begin to compare various species (including sauropsids and primates) in which the dorsal cortex has a less or more complex layering pattern with different levels of cellular diversity and complexity.

## Experimental Procedures

### Dissections and Sequencing

Eight adult male mice (56 days old; C57BL/6J strain) were killed by cervical dislocation according to approved schedule one UK Home Office guidelines (Scientific Procedures Act, 1986). The eight comprised two groups of four littermates each. The mice were decapitated, the skull was opened down the midline, and the brain was removed. Newly dissected brains were rinsed in RNase-free PBS, submerged in ice-cold RNAlater (Ambion) for 24 hr, and stored at −20°C in RNAlater (Ambion). Whole brains were embedded in 5% agarose and sectioned with a vibrating microtome (Leica, VT1000S) into 200 μm coronal sections with a 1:1 mixture of RNAlater and PBS. Six sections corresponding approximately to cortical layers 1–3, 4, upper 5, lower 5, 6, and 6b (henceforth referred to as samples A–F, respectively) were dissected out under visual guidance, with transillumination on a dissecting microscope (MZFLIII, Leica) and stored separately in RNAlater at −80°C until all microdissection was complete. For RNA extraction, samples from individual zones from the eight mice were combined and all tissue samples were processed concurrently. For providing a biological replicate sample, the RNA for sample B was extracted and pooled by litter, providing two samples each representing four mice (heretofore known as samples B1 and B2). Total RNA >200 nt was extracted with the RNeasy Lipid Tissue Mini kit (QIAGEN), in accordance with the manufacturer's instructions and with the on-column DNase digest. RNA quantity was assessed using a NanoDrop 1000 spectrophotometer (ThermoScientific), and RNA quality and integrity assessed using a BioAnalyzer (Agilent Laboratories) (see also Extended Experimental Procedures). Both ends of cDNA fragments corresponding to poly(A) RNA were deep sequenced on Illumina's Genome Analyzer IIx (see Supplemental Experimental Procedures). Sequence reads were mapped to the mouse genome, including splice sites, with TopHat (Trapnell et al., 2009), and de novo transcript models were built and quantified, along with known genes, with cufflinks (Trapnell et al., 2010) as described in the Supplemental Experimental Procedures.

### Classifier Training and Validation

For removal of low-quality quantifications and improve predictions, the de novo transcript models, de novo gene models, Ensembl transcript models, and Ensembl gene models were only used in classification if the width of the largest 95% confidence interval of expression quantification among the samples was less than or equal to 50% the average FPKM across libraries. This retained 11,410 (34%) Ensembl genes (release 57) and 10,261 (45%) Ensembl protein-coding genes.

Manually annotated layer enrichments for genes (matched for strain, sex, age, and cortical region: http://mouse.brain-map.org/pdf/SomatosensoryAnnotation.xls) were processed as described in the Supplemental Experimental Procedures. In total, 2,200 “classifiable” Ensembl genes were included in at least one of these sets. For each individual layer 2/3–6b and “no layer enrichment,” the interactive software package Orange (Demšar et al., 2004) was used for training a naive Bayes classifier to assign, for all genes, the probability that a gene was enriched in the layer of interest, which was subsequently calibrated (Supplemental Experimental Procedures). No “model selection” step was necessary for the naive Bayes classifiers, given that there were no user-adjustable parameters to optimize. Hence, classifier metrics based on 10-fold cross-validation are expected to generalize when applied more broadly to expression distributions across samples of genes and transcripts. Although this does not necessarily require that genes in the Allen Mouse Brain Atlas are representative of cortex-expressed genes on the whole, it does assume that the average correspondence between apparent layer distributions derived from in situ hybridizations and gene expression distributions across samples derived from RNA-seq is similar for Allen Mouse Brain Atlas-curated genes and our classifiable genes and transcripts. So long as this average is consistent, specific deviations (which may arise from complex patterns of expression across diverse cell types, in which quantitative expression measurement may not match qualitative distribution measures) are accounted in the classifier's measures of accuracy (see Belgard et al., 2011 for a notable exception).

### Classifier Predictions of Genes and Transcripts

Once trained and validated, classifiers were applied to the laminar expression distributions of known and de novo genes and transcripts that met the criteria for classification as described above. A single Ensembl gene was considered to have alternatively spliced variants that are differentially expressed across layers if all the following conditions were met:

1. In at least one pair of sequenced samples, the 95% confidence intervals of FPKM expression of a transcript of this gene (as calculated by cufflinks) must not overlap, indicating higher expression in one sample. Another transcript of this gene must additionally have nonoverlapping 95% confidence intervals for the same two samples that indicate higher expression in the opposite sample. Of the 2,003 classifiable genes (17 receptors or ion channels), this retained 1,646 (82%), of which 14 encoded receptors or ion channels.
2. The Euclidean distance in calibrated layer enrichment probability space (square root of the sum of the squares of the differences of layer enrichment probabilities) was greater than 0.5 for at least one pair of its isoforms. Of the 1,646 classifiable genes showing significant differences in expression across samples, this retained 719.

### Functional Differences

We looked for two types of functional difference in our data: (1) functions enriched or depleted in genes predicted to be patterned across layers as compared to genes predicted to be evenly expressed and (2) functions enriched in genes expressed in a specific layer as compared to the set of all classifiable genes. We addressed these two questions in different ways owing to the complicating nature of the classifiers.

The first type of functional difference was based on a two-sided Fisher's exact test comparing the predicted set of patterned genes (all genes predicted to be patterned by at least one classifier) and the predicted set of unpatterned genes. The null hypothesis of each term-wise test is that there is no difference in the proportion of genes with that term between the patterned and unpatterned sets of genes. A test was only made for a term if it was sufficiently powered to detect the maximal possible difference at a p value < 0.05, given the frequency of that term in the union of patterned and unpatterned sets. The R package fisher test was used. For “conditional” databases (mouse knockout phenotypes [Blake et al., 2011], GO [Ashburner et al., 2000]), the 2 × 2 contingency table was only constructed with genes having at least one annotation in that database. For nonconditional databases (KEGG [Kanehisa et al., 2004] molecular pathways, mouse orthologs of human genes nearby SNPs associated with phenotypes by the Ensembl Variation database [Chen et al., 2010], GO [Ashburner et al., 2000] biological process and molecular function), all genes were used for constructing the contingency table.

The second type of functional difference was based on a simulated null distribution. The null hypothesis of each term-wise test is that there is no difference in the proportion of genes with that term between the genes with enriched expression in the layer being considered and all cortex-expressed classifiable genes. Briefly, for each layer, genes were simulated with replacement from the set of all classifiable genes with a probability reflecting the precision of the classifier for that layer. Otherwise, genes were selected with replacement from one of the predicted sets with a likelihood that would best simulate the quantified sources of false positives for that classifier (see Supplemental Experimental Procedures). This continued until the number of simulated genes matched the number of genes in the predicted set (or the number of genes associated with a term in that functional database, for conditional databases). p values for the one-sided test were empirically determined from 200,000 such simulations for every term included in the background distribution, including those terms having no p value in the foreground. This was also done for genes predicted to have no layer enrichment.

To account for multiple testing, q values (which reflect the smallest false discovery rate at which a term would be significant) were calculated from empirical p values with QVALITY v1.11 (Käll et al., 2008) on a per-database basis for both types of functional comparisons. Enrichments not meeting a q value threshold of 0.05 were discarded, controlling false positives at or below 5%.

### Identification of Cortical LincRNAs

We identified in our cortical transcript set 4,587 multiexonic intergenic transcripts with no overlap with Ensembl protein-coding gene annotations (gene build 59). Cortical transcripts with one or more exonic base overlapping an Ensembl protein coding gene exon were used for expanding that gene for purposes of defining intergenic space (Ponting and Belgard, 2010). We calculated, in both orientations (forward and reverse), the coding potential of all intergenic transcripts using the coding potential calculator (Kong et al., 2007) and identified 1,879 intergenic noncoding transcripts longer than 200 bp (lincRNAs). These lincRNAs can be clustered into 1,055 lincRNA loci, defined as the set of transcripts that share at least one intronic or exonic base on either strand.

### In Situ Hybridization

A 982 bp region of *Anxa5* (ENSMUSG00000027712) matching probe RP_040324_01_D04 (Lein et al., 2007) and a 520 bp region of its associated lincRNA (*Gm11549*) matching probe RP_060220_05_F09 (Lein et al., 2007) were separately PCR amplified and cloned into the pCR4-TOPO vector (Invitrogen). P56 C57BL/6 male mouse brains were frozen in OCT (Merck, Darmstadt, Germany) on dry ice, and 14 μm coronal cryosections were cut and mounted on positively charged slides. Digoxigenin-labeled riboprobe synthesis and hybridization were performed as described previously (Isaacs et al., 2003). Sense strand riboprobe hybridization generated no detectable signal in all cases (data not shown). Slides were exposed for 16 hr.

## Figures and Tables

**Table 1:** Known Genes Classified as Being Patterned across Neocortical Cell Layers

	AUC	Precision	Recall	Genes Predicted	Genes Expected
Layers 2/3	0.72	44%	42%	2598	2722
Layer 4	0.88	35%	53%	729	481
Layer 5	0.76	61%	61%	3120	3120
Layer 6	0.77	38%	41%	618	573
Layer 6b	0.75	39%	40%	1104	1076
Unpatterned	0.66	49%	50%	5689	5575

Three statistics from each classifier are presented: *a*rea *u*nder the receiver operating characteristic *c*urve (AUC), the proportion of genes with known expression patterns classified as highly expressed that were previously documented to have elevated expression in the layer (“precision”), and the proportion of the total genes documented to have elevated expression in the layer that are correctly identified by the classifier as highly expressed in that layer (“recall”). The next column contains the number of genes predicted by each classifier out of 11,410 classifiable genes. The last column represents the number of genes expected to be preferentially expressed in this layer, and is calculated as [(number of genes predicted) ^∗^ precision/recall]. The AUC represents the probability that the classifier will rank a randomly chosen gene that is preferentially expressed in that layer above a randomly chosen gene that is not preferentially expressed in that layer: a random classifier has an expected AUC of 0.5. Layer enrichment probabilities of these genes, calibrated as shown in Figure S2, are provided in Table S2.

**Table 2:** Predicted Gene Sets for Various Layers Are Similar in a Replication from an Overlapping Cortical Area and Also from a Nonoverlapping Cortical Area

Classifier	Overall Concordance	Enriched Concordance	AUC	Precision	Recall	Genes Predicted	Genes Expected
**Dorsal Cortex Replication**							

Layers 2/3	79%	57%	0.73	42%	52%	3290	2657
Layer 4	96%	50%	0.83	44%	27%	274	447
Layer 5	74%	62%	0.76	59%	67%	4258	3750
Layer 6	96%	61%	0.78	37%	37%	884	884
Layer 6b	93%	58%	0.74	40%	39%	1200	1231
Unpatterned	67%	68%	0.68	52%	51%	6434	6560

**Lateral Cortex Replication**							

Layers 2/3	78%	55%	0.68	38%	44%	3279	2832
Layer 4	96%	51%	0.85	42%	30%	405	567
Layer 5	71%	55%	0.70	56%	60%	4178	3899
Layer 6	94%	48%	0.83	42%	52%	1001	809
Layer 6b	91%	52%	0.77	41%	46%	1375	1226
Unpatterned	64%	65%	0.63	46%	43%	6144	6573

*Overall concordance* is calculated as (# genes predicted enriched in both + # genes predicted not enriched in both) / (total # genes with a prediction in both). Much of the variability in the overall concordance is due to class imbalance (that is, fewer genes are predicted to be enriched in both original and replication data sets in layer 4 than in layer 5). *Enriched concordance* is calculated as [# genes predicted enriched in both/(# genes predicted enriched in both + # genes predicted enriched in original set only) + # genes predicted enriched in both/(# genes predicted enriched in both + # genes predicted enriched in replication set only)]/2. This average is reported to account for unequal precision and recall between original and replication classifiers. *AUC*, *precision*, *recall*, *genes predicted* and *genes expected* are defined in the Table 1 legend. A total of 12,880 genes were predicted in the dorsal cortex replication, overlapping 9,690 genes classified in the original data set. Similarly, 12,889 genes were predicted in the lateral cortex set, overlapping 9,700 genes classified in the original data set. We estimated 49% of genes were patterned in both the dorsal and lateral replications, similar to the 51% in the original data set. Functional enrichments were generally replicated in both cortical areas with these gene sets (Table S6). Finally, although the lateral cortex was generally concordant with the dorsal cortex, we also identified numerous genes that differed in laminar patterning across areas (Figure S6).

## Accession Numbers

All raw data are also available in GEO and the SRA under accession number GSE27243.

## Supplemental Information

Document S1. Supplemental Experimental Procedures, Six Figures, and Tables S1 and S5

Table S2. Layer Enrichment Probabilities of Known Genes Related to Table 1. Calibrated and uncalibrated layer enrichment probabilities of Ensembl (release 57) genes are shown. For inclusion in the set of predicted layer-enriched genes for functional analysis, the uncalibrated enrichment probability (on which classifier metrics were based) for that layer was required to be greater than 0.5. Layer enrichment probabilities of de novo genes and transcripts, as well as FPKM values across samples for all genes and transcripts, are available from Belgard et al. (2011).

Table S3. Layer Enrichment Probabilities of Genes Encoding Receptors and Ion Channels Related to Figure 2. Calibrated and uncalibrated layer enrichment probabilities of genes encoding known receptors and ion channels (list curated from www.iuphar-db.org and the literature). Italicized gene name and ID indicates no layer predictions were available for that gene. This table also includes qualitative descriptors of consistency or inconsistency with layer enrichment patterns apparent in the Allen Mouse Brain Atlas and links to the relevant images for genes used in Figure 2B. Note that where a gene is enriched in noncontiguous layers, the larger enrichment is more likely to be picked up than the more subtle one. Layer enrichment probabilities for transcripts of these genes, where alternatively spliced, are provided in Table S4.

Table S4. Layer Enrichment Probabilities of Known Transcripts, Highlighting Alternatively Spliced Isoforms Related to Figure 3. Layer enrichment probabilities of transcripts, including those emanating from alternatively spliced Ensembl (release 57) genes (where at least two of the transcripts could be classified) where genes are sorted by the largest Euclidean distance in calibrated layer enrichment probability space between any of the annotated isoforms for that gene are shown. Alternatively spliced transcripts encoding receptors and ion channels (list curated from www.iuphar-db.org and the literature) are also specifically provided. Shorter lists of alternatively spliced transcripts were filtered by requiring differential expression of at least two AS transcripts in opposite directions amongst sequenced samples (see Experimental Procedures).

Table S6. Replication in Dorsal and Lateral Cortex of Functional Terms that Were Significantly Different in the Original Set, and Distribution of Genes in Figure 4 in Dorsal and Lateral Cortex and in the Allen Mouse Brain Atlas Related to Table 2. We also compared the in situ hybridization curations to the layer-wise functional enrichments in Figure 4. None of the five was significantly less enriched in the in situ hybridization curations compared to S1, and the Parkinson's disease enrichment in layer 5 was significantly even more enriched than it was in S1 (p < 0.0001; two-tailed Fisher's exact test). However, some of these tests were underpowered because the Allen Mouse Brain Atlas curations generally had far lower coverage of these gene sets than we did.

Table S7. Layer Enrichment Probabilities of Patterned LincRNA Transcripts Related to Figure 5. Calibrated and uncalibrated layer enrichment probabilities of 76 patterned lincRNA transcripts emanating from 66 patterned lincRNA loci.
