# Supplementary material for: A Transcriptomic Atlas of Mouse Neocortical Layers
Source: Neuron. 2011 Aug 25;71(4-26):605–16. doi: 10.1016/j.neuron.2011.06.039 (PMC3163272; doi:10.1016/j.neuron.2011.06.039)
Supplement: Document S1. Supplemental Experimental Procedures, Six Figures, and Tables S1 and S5 [file mmc1.pdf]

Neuron, *71*

Supplemental Information

A Transcriptomic Atlas of Mouse Neocortical Layers

T. Grant Belgard, Ana C. Marques, Peter L. Oliver, Hatice Ozel Abaan, Tamara M. Sirey, Anna Hoerder-Suabedissen, Fernando García-Moreno, Zoltán Molnár, Elliott H. Margulies, and Chris P. Ponting

Supplemental Data

Figure S1, related to Figure 1. Classifier ROC (receiver operating characteristic) curves for classifiers of all genes.

ROC curves with error bars for each of the classifiers (A) layers 2/3, (B) layer 4, (C) layer 5, (D) layer 6, (E) layer 6b and (F) no layer enrichment. The diagonal with a slope of 1 represents the expectation value of a random classifier. Error bars indicate sample standard deviations based on 10-fold cross validation.

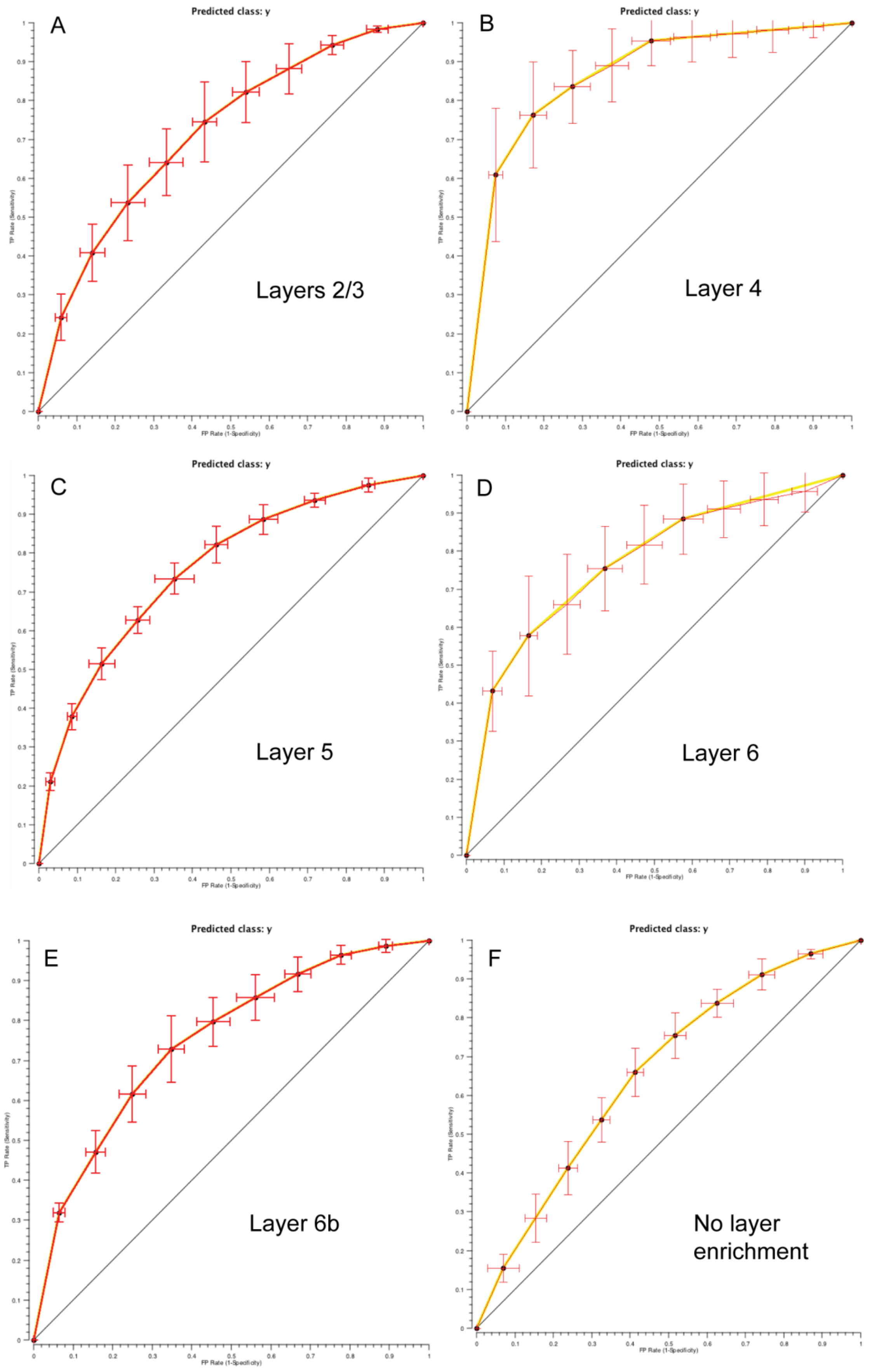
A
Predicted class: y
Layers 2/3
B
Predicted class: y
Layer 4
C
Predicted class: y
Layer 5
D
Predicted class: y
Layer 6
E
Predicted class: y
Layer 6b
F
Predicted class: y
No layer
enrichment
TP Rate (Sensitivity)
FP Rate (1-Specificity)

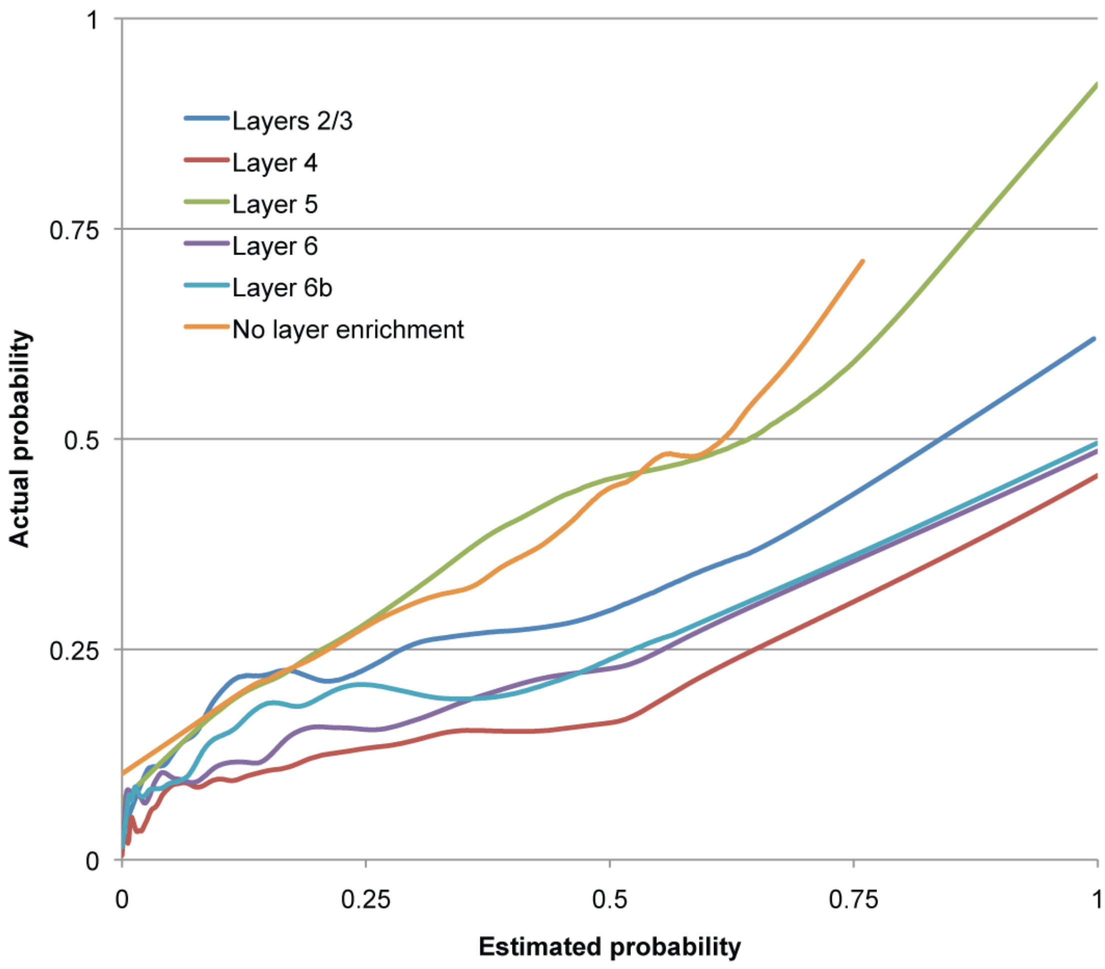

Figure S2, related to Table 1. Probability calibration curves for all genes. LOESS (locally weighted scatterplot smoothing) probability calibration curves comparing the uncalibrated "estimated probability" assigned by the classifier with the cumulative "actual probability" based on the probability values of genes of known layer-enrichment for each of the layer classifiers.

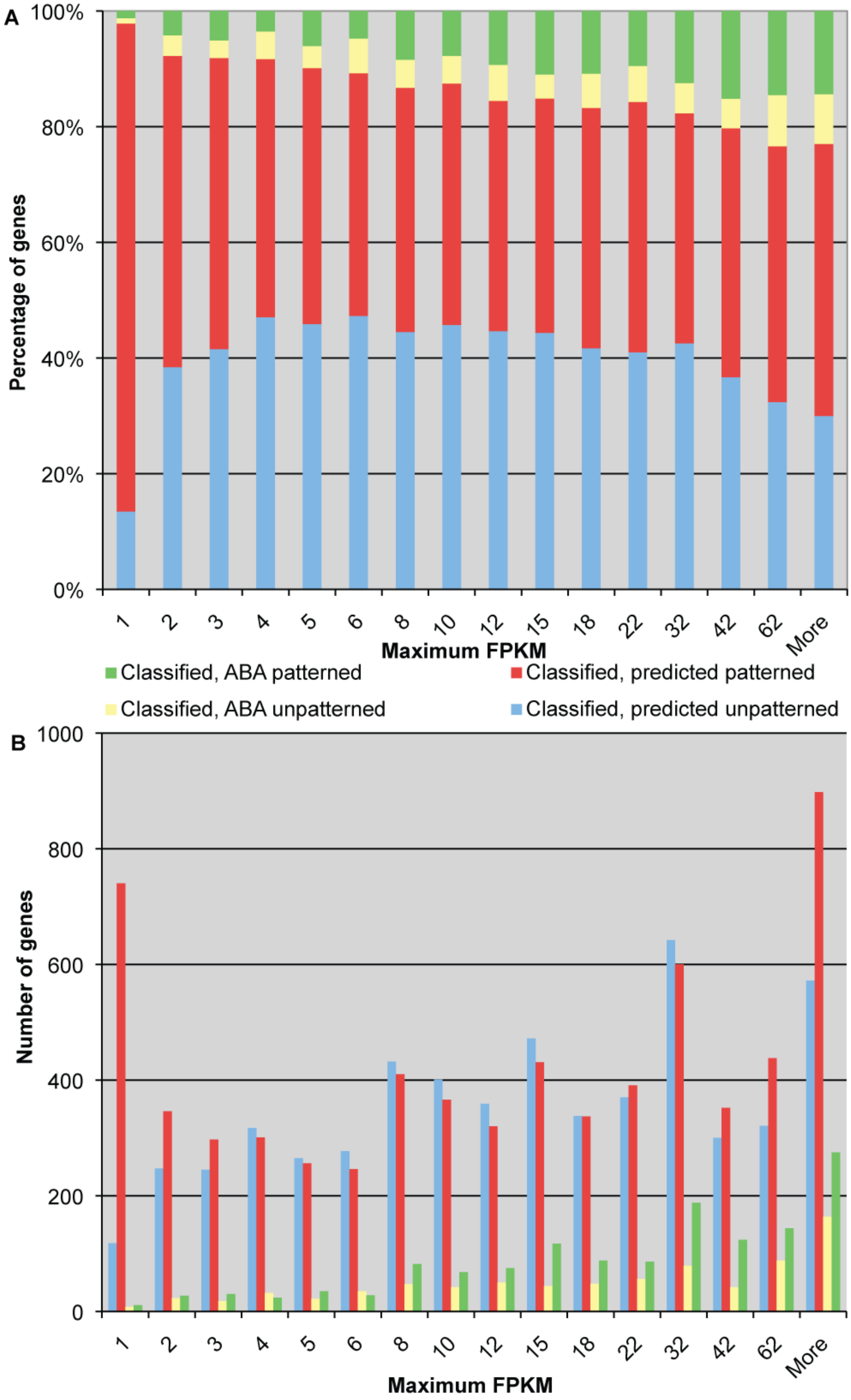
A
Percentage of genes
100%
80%
60%
40%
20%
0%
1 2 3 4 5 6 8 10 12 15 18 22 32 42 62 More
Maximum FPKM
Classified, ABA patterned
Classified, predicted patterned
Classified, ABA unpatterned
Classified, predicted unpatterned
B
Number of genes
1000
800
600
400
200
0
1 2 3 4 5 6 8 10 12 15 18 22 32 42 62 More
Maximum FPKM

Figure S3, related to Figure 2. High-throughput *in situ* hybridization curations cover relatively few genes for which we have RNA-seq-based layer classifications, especially among genes expressed at low levels.

(A) Relative proportions and (B) absolute numbers of genes having different maximum FPKM values across dissected samples. Bin labels give the ceiling for that bin. For a discussion on the outsized number of genes predicted to be patterned at <1 FPKM, see the Online Supplementary Notes (website). Out of 1,780 Allen Mouse Brain Atlas manually curated patterned genes we translated, 1,402 (79%) were classifiable. At the time of writing, a full 2,072 genes were manually curated as being patterned by the Allen Mouse Brain Atlas. With 6,734 predicted patterned genes, we thus increase the number of known layer-patterned genes by 3.25 [6,734/2,072] to 4.25 fold [(6,734+2,072)/2,072].

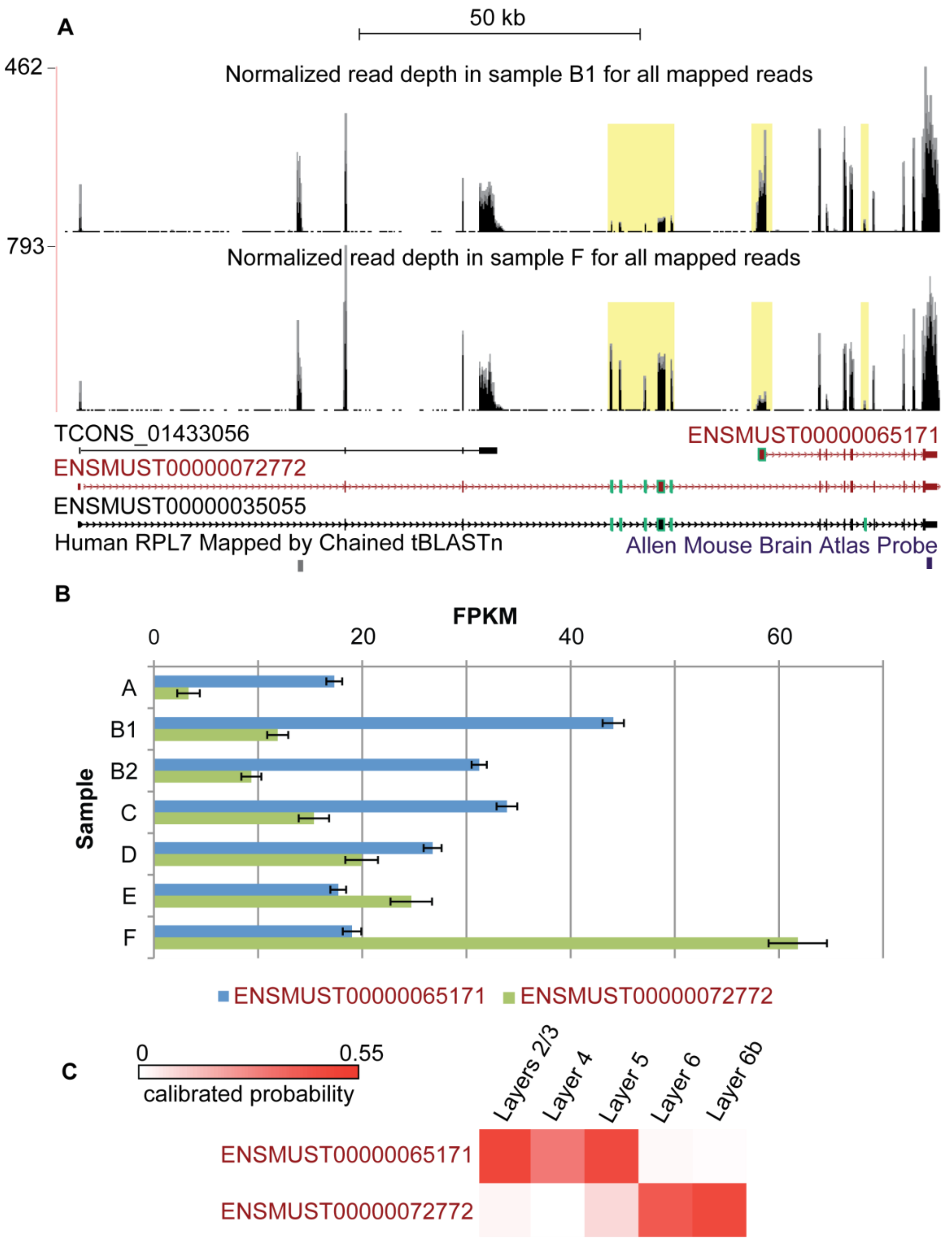

Figure S4, related to Figure 3. *Mtap4* shows signs of differential expression of its isoforms across layers.

Mtap4, the most connected hub gene in Alzheimer's disease (Ray et al., 2008), encodes isoforms of MAP4 with differing microtubule-stabilization properties (Hasan et al., 2006) that have been proposed to regulate the dynamic behaviors of extending neurites (Hasan et al., 2006). (A) Normalized read depth across samples A-F for the

mouse *Mtap4* region (chr9:109,830,555-109,986,919) shows differential expression of ENSMUST00000065171 and ENSMUST00000072772. The full-length transcript, ENSMUST00000035055 (encoding UniProt protein P27546, the canonical mouse MAP4), was a minor isoform in comparison, and *de novo* transcript TCONS_01433056 lacks the MAP4 microtubule-binding domain. In contrast, the two differentially expressed transcripts marked in red, ENSMUST00000065171 (encoding P27546 isoform 4) and ENSMUST00000072772 (encoding P27546 isoform 3), encode known proteins that differ in sequence. Isoform 3 contains one fewer Tau/MAP domain than isoform 4. The number of these domains is known to affect kinesin motor activity on microtubules (Tokuraku et al., 2007). Yellow regions mark the exons, boxed in green, that discriminate among isoforms. The reads mapping to the region with similarity to human RPL7 likely derive from one of many retropseudogenes of the mouse *Rpl7* gene, which is highly expressed in all cortical samples. The probe used for *in situ* hybridization in the Allen Mouse Brain Atlas does not discriminate between these isoforms. (B) FPKMs across samples with 95% confidence intervals suggest the two highly-expressed and potentially functional isoforms are differentially patterned across samples. (C) Classifiers also predict that these two isoforms are strongly differentially patterned across layers.

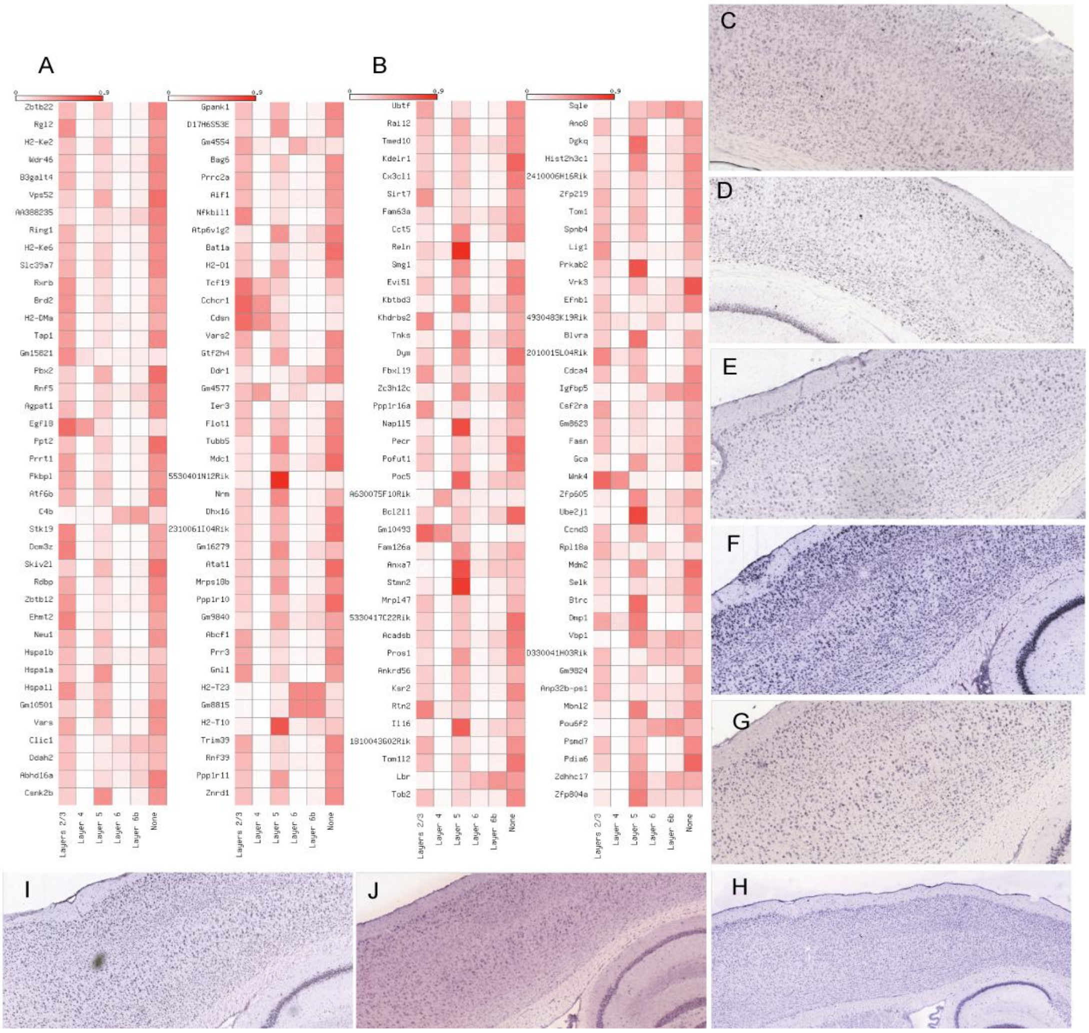

Figure S5, related to Figure 4. Many MHC region genes show enriched expressed in layers 2/3.

(A) Heatmaps (Pavlidis and Noble, 2003) representing calibrated layer enrichment probabilities of MHC region genes in order of their leftmost coordinate, ordered from top to bottom, then left to right. Genes show complex patterns of enrichment across layers. (B) Heatmaps representing calibrated layer enrichment probabilities for 80 genes selected at random using a pseudorandom number generator from the list of classified genes. MHC region genes are 34% more likely than these randomly selected genes to be enriched in layers 2/3 (0.302 vs 0.226 average calibrated probabilities of enrichment; $p < 10^{-6}$, case resampling bootstrap). We display *in situ*s

from the Allen Mouse Brain Atlas (AMBA) of some example genes that appear correctly predicted by our classifiers as being enriched in at least layers 2/3 (they may also be enriched in other layers): (C) *Ier3* (AMBA curators confirm 2/3, also report 6), (D) *Hspa1l* (AMBA curators confirm 2/3, also report 6 and 6b), (E) *H2-DMa* (however AMBA curators report none), (F) *Dom3z*, (G) *Ehmt2*, (H) *Cchcr1*, (I) *Stk19* and (J) *Egfl8*. *Tcf19* is an example of a gene unresolved in most coronal sections (expression appears even across everything in brain), but at least one section has a clean stain (this stain shows a layers 2/3 enrichment). *Cdsn*, another gene with high likelihood of layers 2/3 enrichment, is untried at the time of this writing. Such high-level descriptive findings highlight the advantages of a genomics approach to neuroanatomy.

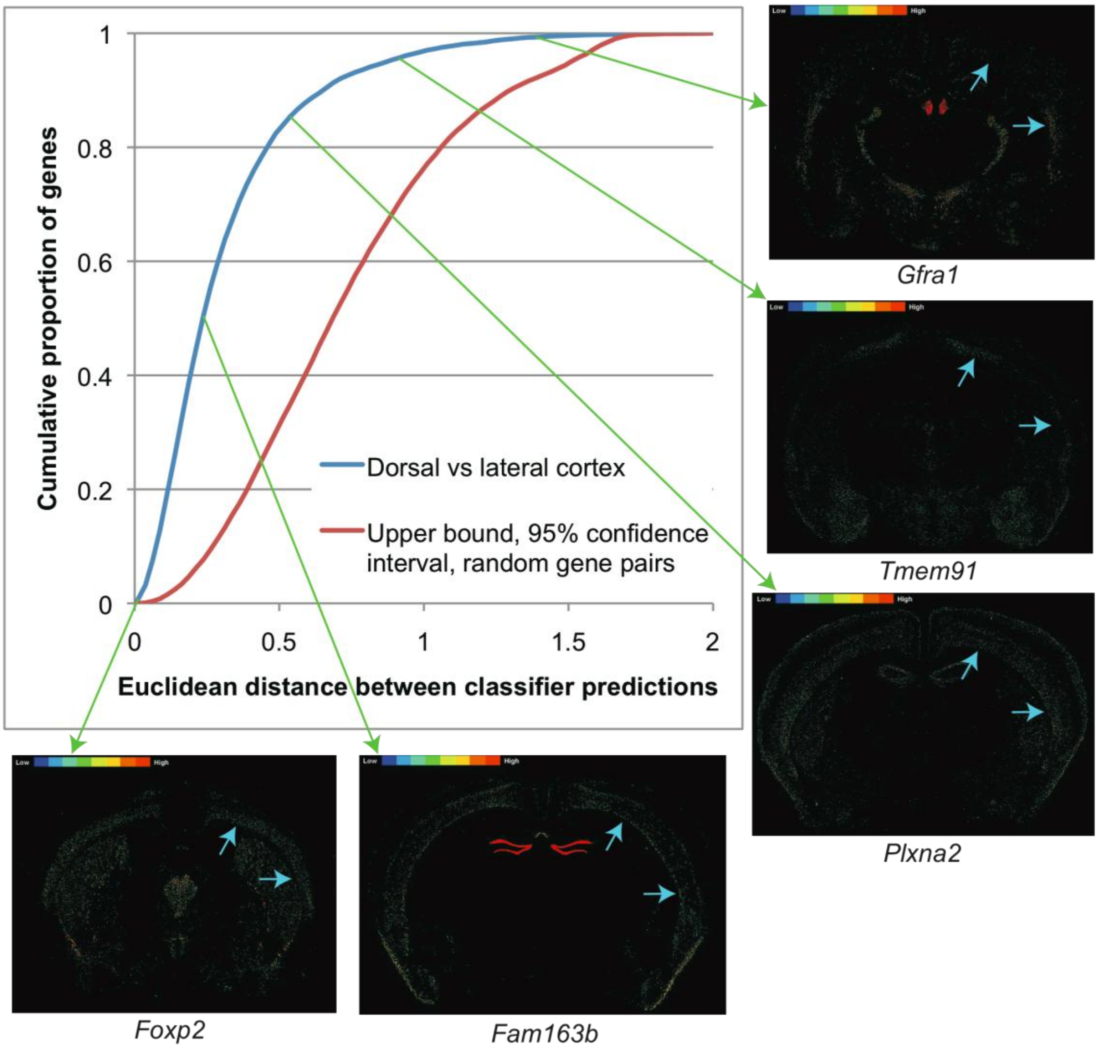

Figure S6, related to Table 2. Most, but not all, genes have a similar predicted laminar expression pattern in both dorsal cortex and lateral cortex.

In blue, the cumulative frequency distribution of the Euclidean distances between laminar enrichment probabilities of genes in lateral cortex and the dorsal cortex replication demonstrates that most genes have similar predictions in both regions. Most genes have a Euclidean distance smaller than 0.25, suggesting their predicted laminar expression pattern is similar between dorsal and lateral cortex. The red distribution represents the upper bound of the one-tailed 95% confidence interval obtained by randomly pairing genes. This was calculated as follows: 500 distributions were bootstrapped by randomly pairing, with replacement, layer predictions in lateral cortex and in the dorsal cortex replication from 12,455 genes;

these distributions were sorted by numerically integrating over Euclidean distances 0 to 2, inclusive, and the resulting 95th percentile distribution is displayed. The relative positioning of these two distributions quantitatively grounds the qualitative observation that a difference in laminar expression patterns between dorsal and laminar cortices is the exception to the rule. If, instead, there were no relationship between expression patterns in dorsal and laminar cortex, the laminar cortex classifiers (trained on curations based on dorsal cortex) would have poor AUC values and one would instead expect the blue curve to lie beneath the red curve. Gene expression analysis of the coronal *in situ* hybridization images from the Allen Mouse Brain Atlas for representative genes on this continuum: *Foxp2* (0.01), *Fam163b* (0.25), *Plxna2* (0.55), *Tmem91* (0.9), and *Gfra1* (1.4). The upper teal arrow indicates dorsal cortex, while the lower teal arrow indicates lateral cortex. Comparing laminar expression patterns in dorsal and lateral cortices, *Foxp2* and *Fam163b* show no differences (both genes show enrichment in deeper layers in each area), *Plxna2* shows slight differences (enriched in deeper layers in both areas, but slightly more so in laminar cortex), and *Tmem91* (heavy expression in layer 5 of dorsal cortex, sparse and relatively unpatterned expression in lateral cortex) and *Gfra1* (heavy expression in deep layers of lateral cortex, sparse and unpatterned expression in dorsal cortex) show large differences.

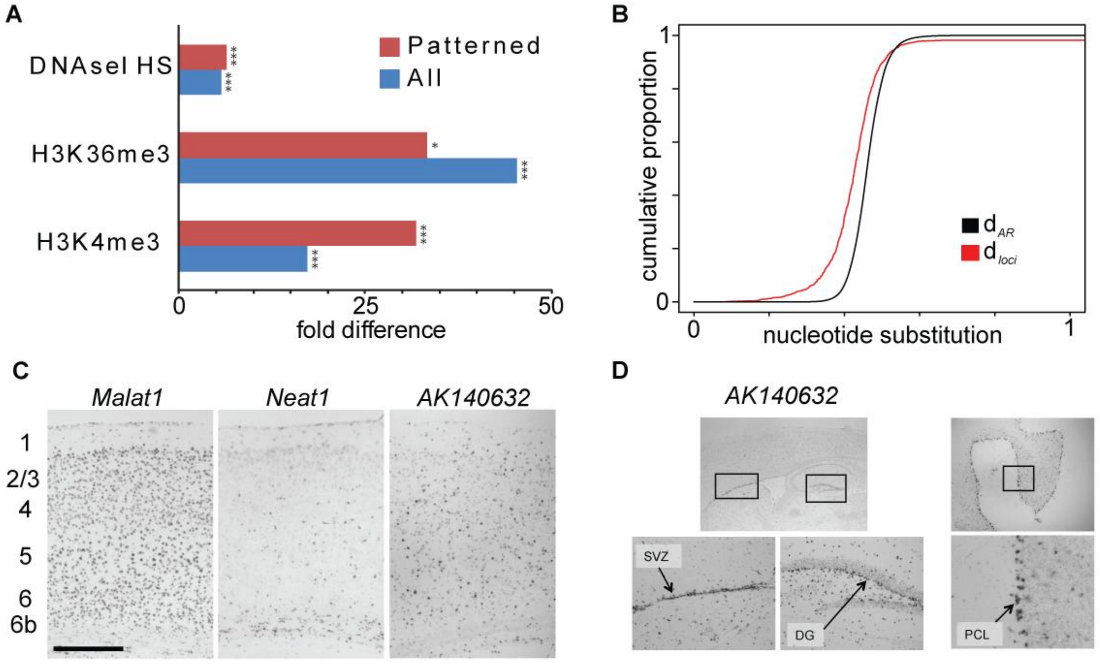

Figure S7, related to Figure 5. LincRNAs are transcribed, evolutionarily constrained, and patterned in expression.

(A) LincRNA loci significantly coincide with DNAseI hypersensitivity sites and histone methylation marks associated with active transcription in neuronal precursor cells. Plot represents fold enrichments of observed over expected values for the overlap of layer patterned lincRNA loci and all lincRNA loci with DNAseI hypersensitivity sites, H3K36me3 sites and H3K4me3 sites (Meissner et al., 2008; Mikkelsen et al., 2007). *$p < 0.05$, ***$p < 0.001$ (B) LincRNA loci show significant evolutionary constraint. Plot represents the cumulative distributions of substitution rate for lincRNA loci (red) and putatively neutral sequence-AR (black). LincRNA loci accumulate significantly fewer nucleotide substitutions between mouse and human than neighboring neutral sequence (0.428 and 0.460: median substation rate lincRNA loci and neutral sequence respectively; $p < 10^{-16}$). (C) Some cortical lincRNAs are bona fide layer markers. In situ hybridization of three lincRNAs in the male mouse cortex at P56. *Malat1* is the among the most highly expressed transcripts

identified from the RNA-seq dataset. *Neat1* expression is highly patterned in layer 6b, as predicted, although in these images *AK140632* is expressed at low levels throughout the cortex. Scale bar is 500 μm. (D) Some cortical lincRNAs are expressed more highly elsewhere in the brain. *AK140632* is also expressed in a specific subset of neurons in adult brain as shown from parasagittal adult brain sections, including the dentate gyrus (DG), subventricular zone (SVZ) and the nuclei of neurons in the Purkinje cell layer (PCL) of the cerebellum.

Sample	Bases (% genome) covered by at least one read	Bases (% genome) covered by at least two non-identical reads	Bases (% intergenic genome) outside non-lincRNA Ensembl loci covered by at least one read	% Ensembl genes >0.0 FPKM	% Ensembl genes >0.1 FPKM
A	268040319 (9.8%)	160004371 (5.9%)	56,436,638 (3.3%)	63%	54%
B1	290720044 (10.7%)	148490006 (5.4%)	61,498,352 (3.6%)	61%	50%
B2	372478215 (13.7%)	239652510 (8.8%)	84,657,473 (5.0%)	64%	51%
C	259025712 (9.5%)	138128102 (5.1%)	53,839,576 (3.2%)	61%	51%
D	236340872 (8.7%)	129833188 (4.8%)	50,051,652 (2.9%)	61%	51%
E	238855138 (8.8%)	126055098 (4.6%)	48,854,567 (2.9%)	62%	51%
F	211938161 (7.8%)	145046680 (5.3%)	42,521,967 (2.5%)	60%	52%
Merged	671356672 (24.6%)	365230720 (13.4%)	170,720,024 (10.0%)	71%	59%

Table S1, related to Figure 1. Coverage statistics of the genome, known genes, and intergenic regions.

Bases (% genome) covered by at least one read represents the number of bases and proportion of the 2,725,765,481 bases in mm9 used in these alignments (all chromosomes, including randoms, using the UCSC nomenclature, and chrM). *Bases (% genome) covered by at least two non-identical reads* represents the number & proportion of such bases covered by two non-identical reads, where "samtools rmdup" has been run on the bam files to remove paired-end reads that have the exact same start and stop mapped locations (depleting PCR and optical duplicates). *Bases (% intergenic genome) outside non-lincRNA Ensembl loci covered by at least one read* represents the number of bases, and proportion of the intergenic genome (1,704,768,106 nt remains after eliminating the 37.5% of the mouse genome inside non-lincRNA genic loci), covered by at least one read outside all loci of all classes of

genes defined in Ensembl release 60, excepting lincRNAs. *% Ensembl gene loci >0.0 FPKM* represents the percentage of all Ensembl genes (release 57, which did not include lincRNA gene predictions) with any nonzero FPKM expression value called by cufflinks (see Extended Experimental Procedures). Among protein-coding genes in particular, 18,960 genes (83% of the 22,806 total) had nonzero FPKMs in at least one sample. *% Ensembl genes >0.1 FPKM* represents the same as before but for genes greater than 0.1 FPKM. 16,340 protein-coding genes (72%) were expressed at greater than 0.1 FPKM in at least one sample.

(TableS2.xls)

Table S2, related to Table 1. Layer enrichment probabilities of known genes.

Calibrated & uncalibrated layer enrichment probabilities of Ensembl (release 57) genes. To be included in the set of predicted layer-enriched genes for functional analysis, the uncalibrated enrichment probability (on which classifier metrics were based) for that layer was required to be greater than 0.5. Layer enrichment probabilities of *de novo* genes and transcripts, as well as FPKM values across samples for all genes and transcripts, are available from the website.

(TableS3.xls)

Table S3, related to Figure 2. Layer enrichment probabilities of genes encoding receptors and ion channels.

Calibrated and uncalibrated layer enrichment probabilities of genes encoding known receptors and ion channels (list curated from www.iuphar-db.org and the literature). Italicized gene name and ID indicates no layer predictions were available for that gene. This table also includes qualitative descriptors of consistency or inconsistency with layer enrichment patterns apparent in the Allen Mouse Brain Atlas and links to

the relevant images for genes used in Figure 2B. Note that where a gene is enriched in non-contiguous layers, the larger enrichment is more likely to be picked up than the more subtle one. Layer enrichment probabilities for transcripts of these genes, where alternatively spliced, are provided in Table S4.

(TableS4.xls)

Table S4, related to Figure 3. Layer enrichment probabilities of known transcripts, highlighting alternatively spliced isoforms.

Layer enrichment probabilities of transcripts, including those emanating from alternatively spliced Ensembl (release 57) genes (where at least two of the transcripts could be classified) where genes are sorted by the largest Euclidean distance in calibrated layer enrichment probability space between any of the annotated isoforms for that gene. Alternatively spliced transcripts encoding receptors and ion channels (list curated from www.iuphar-db.org and the literature) are also specifically provided. Shorter lists of alternatively spliced transcripts were filtered by requiring differential expression of at least two AS transcripts in opposite directions amongst sequenced samples (see Experimental Procedures).

Symbol	Name
Uqcrc2	ubiquinol cytochrome c reductase core protein 2
Lrrk2	leucine-rich repeat kinase 2
Atp5c1	ATP synthase, H+ transporting, mitochondrial F1 complex, gamma polypeptide 1
Cox6a2	cytochrome c oxidase, subunit VI a, polypeptide 2
Slc25a4	solute carrier family 25 (mitochondrial carrier, adenine nucleotide translocator), member 4
Ndufv2	NADH dehydrogenase (ubiquinone) flavoprotein 2
Uqcrc1	ubiquinol-cytochrome c reductase core protein 1
Ndufb8	NADH dehydrogenase (ubiquinone) 1 beta subcomplex 8
Uqcrfs1	ubiquinol-cytochrome c reductase, Rieske iron-sulfur polypeptide 1
Ndufa6	NADH dehydrogenase (ubiquinone) 1 alpha subcomplex, 6 (B14)
Ndufb6	NADH dehydrogenase (ubiquinone) 1 beta subcomplex, 6
Cyc1	cytochrome c-1
Sdhd	succinate dehydrogenase complex, subunit D, integral membrane protein
Ndufs1	NADH dehydrogenase (ubiquinone) Fe-S protein 1
Cox5a	cytochrome c oxidase, subunit Va
Sdha	succinate dehydrogenase complex, subunit A, flavoprotein (Fp)
Vdac1	voltage-dependent anion channel 1
Casp3	caspase 3
Atp5f1	ATP synthase, H+ transporting, mitochondrial F0 complex, subunit B1
Vdac2	voltage-dependent anion channel 2
Ube2j1	ubiquitin-conjugating enzyme E2, J1
Ube2g1	ubiquitin-conjugating enzyme E2G 1 (UBC7 homolog, C. elegans)
Ube2g2	ubiquitin-conjugating enzyme E2G 2
Atp5b	ATP synthase, H+ transporting mitochondrial F1 complex, beta subunit
Ppid	peptidylprolyl isomerase D (cyclophilin D)
Ndufa9	NADH dehydrogenase (ubiquinone) 1 alpha subcomplex, 9
Sdhb	succinate dehydrogenase complex, subunit B, iron sulfur (Ip)
Atp5j	ATP synthase, H+ transporting, mitochondrial F0 complex, subunit F
Atp5g3	ATP synthase, H+ transporting, mitochondrial F0 complex, subunit C3 (subunit 9)
Ndufa1	NADH dehydrogenase (ubiquinone) 1 alpha subcomplex, 1
Ndufc1	NADH dehydrogenase (ubiquinone) 1, subcomplex unknown, 1
Ndufc2	NADH dehydrogenase (ubiquinone) 1, subcomplex unknown, 2
Atp5a1	ATP synthase, H+ transporting, mitochondrial F1 complex, alpha subunit 1
Ndufb5	NADH dehydrogenase (ubiquinone) 1 beta subcomplex, 5

Table S5, related to Figure 4. Genes in the Parkinson's Disease enrichment of layer 5.

Nineteen genes with the Parkinson's Disease annotation would have been expected to be enriched in layer 5 by chance. Most of these thirty-four genes encode proteins active or associated with mitochondrial functions.

(TableS6.xls)

Table S6, related to Table 2. Replication in dorsal and lateral cortex of functional terms that were significantly different in the original set, and distribution of genes in Figure 4 in dorsal and lateral cortex and in the Allen Mouse Brain Atlas.

We also compared the *in situ* hybridization curations to the layer-wise functional enrichments in Figure 4. None of the five was significantly less enriched in the *in situ* hybridization curations compared to S1, and the Parkinson's disease enrichment in layer 5 was significantly even more enriched than it was in S1 ($p < 0.0001$; two-tailed Fisher's exact test). However, some of these tests were underpowered because the Allen Mouse Brain Atlas curations generally had far lower coverage of these gene sets than we did.

(TableS7.xls)

Table S7, related to Figure 5. Layer enrichment probabilities of patterned lincRNA transcripts.

Calibrated and uncalibrated layer enrichment probabilities of 76 patterned lincRNA transcripts emanating from 66 patterned lincRNA loci.

Supplemental Experimental Procedures

Sequencing, read mapping, transcript building and quantification

Total RNA was prepared for paired-end deep sequencing on Illumina's Genome Analyzer IIx following the manufacturer's protocol. Briefly, poly(A) RNA was enriched using Dynal oligo(dT) beads (Invitrogen). This was fragmented using the RNA fragmentation kit (Ambion). First- and second-strand cDNA was synthesized with random hexamer primers (Invitrogen) and SuperScript II (Invitrogen). Ends were repaired using T4 DNA polymerase and Klenow DNA polymerase. A single adenosine and Illumina adapters were ligated using Klenow 3'-to-5' exo-nuclease. Following gel purification of cDNA templates, the library was enriched with 15 rounds of PCR before being added to the flow cell for paired-end sequencing. 51 nt were sequenced from either end, of which 50 nt were used for mapping for all lanes except two of the five lanes of sample F, in which 76 nt were sequenced and 75 nt were used in mapping. These mixed read lengths had negligible effects on quantifications used in downstream analyses (Online Supplementary Notes on the website).

Reads were processed with versions 1.4 and 1.6 of the Illumina pipeline. The internal insert size and standard deviation were estimated for each library by empirically calculating the full width at half max (FWHM) for an internal insert size

distribution constructed using uniquely mapping reads on the same chromosome from Illumina's Gerald pipeline. The insert size was estimated to be the midpoint of the minimum and maximum insert sizes at half (or greater) the frequency of the most common insert size, and the standard deviation was calculated under a Gaussian assumption StdDev=FWHM/(2*sqrt(2ln2)). Using this insert size and standard deviation, lanes were separately mapped with TopHat v1.0.13 (Trapnell et al., 2009) to the mouse reference genome (mm9, downloaded from UCSC) plus all splice junctions from the UCSC Table Browser (Rhead et al., 2010) tables all_mrna (mouse mRNAs), intronEst (mouse spliced ESTs), and xenoMrna (mRNAs mapped from other species to mouse) with the following options: butterfly search, closure search, fill gaps, microexon search, min anchor length = 5, min isoform fraction = 0.0, max intron length = 500 kb. In a second round of TopHat mapping (default options except min isoform fraction = 0.0), all novel junctions identified from any sample in the previous step were given to TopHat again (as raw junctions), so splice junctions could be measured across all samples in an unbiased manner.

De novo transcript models were built for each sample using CuffLinks v0.8.3 (Trapnell et al., 2010), providing the average insert sizes and standard deviations calculated previously and using default options. Transcript models were combined across all samples using cuffcompare with default options, and comparing against a GTF of Ensembl gene models (Flicek et al., 2011) downloaded from Ensembl (release 57) and adapted to use the UCSC nomenclature. Expression levels of the resultant unified cuffcompare transcript models were assessed in every sample using cufflinks in quantification-only mode with default settings. Expression levels of all genes and transcripts in Ensembl (release 57) were quantified with cufflinks in quantification-only mode with default settings.

Sample	Litter 1 RIN	Litter 2 RIN	Litter 1 concentration (ng/μL)	Litter 2 concentration (ng/μL)	Total amount (μg)
A	3.1	2.4	88.4	132.0	6.6
B	6.6	5.7	310.9	267.0	9.3, 8.0*
C	8.2	6.7	161.0	170.9	10.0
D	8.6	7.6	155.9	152.9	9.3
E	7.8	8.1	156.3	125.2	8.4
F	6.7	8.4	41.9	51.2	2.8

Integrity and quantity of total RNA from dissected samples, by litter.

RNA Integrity Number (RIN) was assessed using a BioAnalyzer and concentration using a NanoDrop 1000 spectrophotometer. Total amount of RNA was estimated by multiplying the concentration by the 30 μL volume remaining after assessing the sample characteristics. *Totals are listed separately for B1 and B2, as they were made into separate libraries.

Sample	Average fragment length	Insert standard deviation	Millions of clusters	Millions of clusters passing filter	Millions of TopHat alignments	Millions of alignable reads	Millions of uniquely alignable reads
A	164	27	60.5	50.8	79.7	74.1	65.7
B1	239	29	67.8	~57.7	116.7	115.0	112.7
B2	193	28	119.4	89.0	186.1	181.9	170.0
C	184	21	63.9	50.8	101.6	99.4	95.2
D	208	22	73.3	59.6	111.9	109.2	105.9
E	172	24	73.6	55.0	104.0	101.4	98.3
F	184	28	71.8	58.3	111.3	109.2	106.8
Combined	-	-	530	421	811	790	755

Fragment sizes and numbers of sequenced and aligned reads per library. Average fragment length was the midpoint of the full width at half maximum (FWHM) of mapped paired ends based on unique ELAND mappings to the mouse genome assembly (mm9); the standard deviation was estimated using a Gaussian assumption (FWHM/2.35). *Millions of clusters* represents the total number of paired end fragments (2x51 (or 2x76) nucleotides, the 51st (or 76th) base was not used in subsequent analysis) imaged across all flowcells. *Millions of clusters passing filter* represents the number of these clusters that passed Illumina's native chastity filter. *Millions of TopHat alignments* represents the number of alignments (in up to 40 genomic locations by default) reported by TopHat, using all clusters as an input (not just those passing the chastity filter). *Millions of alignable reads* represents the number of reads contributing to these alignments. *Millions of uniquely alignable reads* represents these reads that mapped uniquely to the genome or across splice sites.

Normalization and visualization

BigWIG (Kent et al., 2010) and BAM (Li et al., 2009) files were created so that aggregate read coverage and reads, respectively, could be visualized with the UCSC Genome Browser (Rhead et al., 2010). WIG files created by parsing the

output of “samtools pileup” were normalized (such that scales were comparable across samples) as follows, before conversion to BigWIG format. For normalization, uniquely mapping reads were first extracted from alignment files using a custom script and pysam (http://code.google.com/p/pysam/). Then, the number of unique (and uniquely mapping) reads overlapping (at least partially) each Ensembl (release 56) protein-coding gene model were quantified for each sample. The exonic Ensembl models were further required to be >200 nt in size, as the experimental protocol selected against these, and as variations in expression levels might derive from the upstream experimental protocol. Eliminating these 328 small gene models left 22533 Ensembl protein-coding gene models that were used in subsequent analyses.

Read counts were then normalized to adjusted read counts that were directly comparable across samples as a measure of relative expression. For each gene model, the number of reads falling (at least partially) into that model was summed separately for each sample. Then, for each gene model in every sample, the ratio of (read counts in that model in the sample):(total number of read counts in that model across all samples) was calculated. The median ratio across all gene models was found for each sample. Adjusted read counts were calculated for each sample by multiplying all read counts in that sample by (the minimum median ratio of all samples)/(median ratio of that sample). These same ratios were used to adjust WIG coverage densities to (often non-integral) normalized values.

Sets of curated genes

Manually annotated layer enrichments for genes (matched for strain, sex, age, and cortical region) were downloaded from the Allen Mouse Brain Atlas (http://mouse.brain-map.org/pdf/SomatosensoryAnnotation.xls; downloaded 3 June 2010). Gene symbols from distinctive categories containing 11 genes or more were

extracted and mapped to Ensembl gene identifiers using the MGI Batch Query (http://www.informatics.jax.org/javawi2/servlet/WIFetch?page=batchQF). Only those genes having Ensembl identifiers were kept. These categories were then mapped to a smaller number of categories, corresponding to genes enriched in layers 2/3, 4, 5, 6, 6b and genes showing no layer enrichment (or depletion). Corresponding categories were constructed from other genes for the inverse, e.g. "not in 4". For example, genes enriched in layers 2/3 and 5 were marked as enriched in both of these layers. The two negative categories ("not in 4" and "not in 5") were not included in any sets of layer-enriched genes, but only to the sets of genes that were not enriched in layers 4 and 5, respectively.

Microarray comparison

Microarray probes having a fold change greater than 1.5 and a reported *p*-value less than 0.05 were translated to Ensembl gene IDs using the MGI batch query. Relative expression levels of these gene IDs were then compared between samples E and F.

Other classifier considerations

Orange (Demšar et al., 2004) was also used to produce, for each classifier, an expression profile across samples for genes in that layer (Online Supplementary Figure 3 on the website), a pictorial representation of the classification model (Online Supplementary Figure 4 on the website), a Receiver Operating Characteristic (ROC) curve reflecting the sensitivity-specificity tradeoff (Figure S1), and a plot relating the classifier's estimated probability to the actual probability of enrichment based on the training set of 2,200 genes (Figure S2).

Support Vector Machines (SVMs) with radial basis functions were constructed, where C and gamma were chosen to optimize the F_1 score (the harmonic

mean of precision and recall) using leave-one-out cross-validation assessed using a separate test set (15% of the starting set). These had comparable classification metrics to the naïve Bayes models, so the latter were chosen since they provide a per-gene probability score of enrichment as opposed to just a binary classification. Surprisingly, random forest classifiers had comparable performance to naïve Bayes. The random forest classifiers produced more accurate uncalibrated probabilities, but the calibration curves were not as easily modeled as those from the naïve Bayes classifiers.

Classifier probability calibration

We recalibrated the estimated probability produced by the models to the observed probabilities by locally weighted scatterplot smoothing (LOESS) (Cleveland and Devlin, 1988) using the Excel add-in available at http://peltiertech.com/images/2009-10/PTS_LOESS.xla (Figure S2**).** Points corresponded to genes of known laminar patterning (or lack thereof), with the x-coordinate equal to the classifier-produced probability and the y-coordinate either 1 or 0, corresponding to curated enrichment or not, respectively, for that layer. The smoothing parameter, alpha, was 0.33 (726 points in moving regression) for all models except layer 4 and layer 6, where alpha was raised to 0.65 (1430 points) and 0.45 (990 points) respectively, to avoid edge effects.

Expected prevalence of patterned genes

Based on the unpatterned classifier, we expect 5,835 genes to be patterned (11,410-5,575). The sum total of genes expected to be enriched in each of the layers (redundant) is 7,972, which means we expect a gene to be enriched in, on average, 1.37 (7,972/5,835) layers. This is consistent with the 1.38 ([sum of genes enriched in each layer / number of unique genes enriched in any layer] = 2,459/1,780) average

number of layers in which patterned genes we extracted from Allen Mouse Brain Atlas curations (Lein et al., 2007) were expressed, suggesting that, since we would expect these ratios to be similar, extrapolation of total number of patterned genes from the unpatterned classifier statistics is consistent with the layer-specific classifiers. This ratio implies that most of these patterned genes are markers of single layers.

This proportion (51%) is lower than the 62% (1,780/2,861) of genes annotated as being patterned from *in situ* hybridization images (Lein et al., 2007) (Online Supplementary Table 6 on the website). This was unsurprising, as the curated Allen Mouse Brain Atlas gene set was intentionally enriched for likely layer-patterned genes (http://mouse.brain-map.org/pdf/SomatosensoryAnnotationWhitePaper.pdf).

Classical layer markers

Several classical layer markers were taken where a review (Molyneaux et al., 2007) matched Allen Mouse Brain Atlas images taken from primary somatosensory cortex of P56 male black 6 mice (since they were originally curated from a variety of timepoints and cortical areas (Bulfone et al., 1995; Inoue et al., 2004; Lein et al., 2007; Molyneaux et al., 2007; Nieto et al., 2004; Ouimet et al., 1984; Watakabe et al., 2006; Xiong et al., 2004)). All images were from coronal sections (near position 6,000 in the AMBA) except *Kcnip2*, which was from a sagittal section (near position 1,000 in the AMBA).

Annotations used in testing functional differences

All GO (Ashburner et al., 2000) annotations (biological process, molecular function and cellular component) were downloaded from Ensembl BioMart (Smedley et al., 2009) (release 56) and tested independently. For the mouse knockout phenotypes (Blake et al., 2011), Ensembl IDs were translated to Mouse Genome

Informatics (MGI) IDs using data in ftp://ftp.informatics.jax.org/pub/reports/MRK_ENSEMBL.rpt, MGI IDs were translated into low-level phenotypes using data in ftp://ftp.informatics.jax.org/pub/reports/MGI_PhenoGenoMP.rpt and finally associated to all relevant phenotype descriptors using data in ftp://ftp.informatics.jax.org/pub/reports/MPheno_OBO.ontology. Mouse protein complex associations were downloaded from Ensembl BioMart (Reactome database, release 56) (Croft et al., 2011) and tested as a non-conditional database. For pathway information from KEGG (Kanehisa et al., 2004), Ensembl IDs were translated into KEGG gene IDs using data in ftp://ftp.genome.jp/pub/kegg/genes/organisms/mmu/mmu_ensembl-mmu.list (downloaded June 17, 2010), KEGG gene IDs were associated to pathways with data in ftp://ftp.genome.jp/pub/kegg/genes/organisms/mmu/mmu_pathway.list (downloaded June 17, 2010) and pathways were assigned names with the data in ftp://ftp.genome.jp/pub/kegg/pathway/map_title.tab (downloaded June 17, 2010). Miller (Miller et al., 2010) gene co-expression modules in mouse brain were downloaded from http://www.genetics.ucla.edu/labs/horvath/CoexpressionNetwork/MouseHumanBrain/mouse_genes_vs_eigengenes.csv, using the co-expression modules assigned by the authors (Miller et al., 2010) and translated to Ensembl gene IDs using the MGI batch query. Winden (Winden et al., 2009) gene co-expression modules among mouse neurons were downloaded from http://www.ncbi.nlm.nih.gov/pmc/articles/PMC2724976/bin/msb200946-s2.xls, using the co-expression modules assigned by the authors (Winden et al., 2009), and translated to Ensembl gene IDs using the MGI batch query. For the Genome-wide

association study data, we downloaded from BioMart (Ensembl Variation (Chen et al., 2010) release 57; downloaded March 9, 2010) human genes associated with SNPs with significant associations with phenotypes. We removed phenotypes with fewer than ten associated genes. Then we translated these human gene IDs to mouse gene IDs using Ensembl BioMart (keeping only the genes having a one-to-one mapping). In all translations described above, no more than one mapping was taken for any gene to avoid multiple counting.

Simulating the null distributions for assessing functional differences in individual predicted sets

A null distribution was used to assess functions enriched in genes expressed in a specific layer as compared to the set of all classifiable genes. The null hypothesis of each term-wise test is that there is no difference in the proportion of genes with that term between the genes with enriched expression in the layer being considered and all cortex-expressed classifiable genes. To avoid false functional enrichments in a layer due to the presence of false positives from another layer, genes were simulated with replacement as follows:

1. Have a pseudo-random number generator pick a number, x, from 0 to 1. If this number is less than the precision of the set, randomly sample with replacement a gene from all classifiable genes for non-conditional databases, or from the set of all classifiable genes with annotations in this database for conditional databases. We seek the contribution of the true gene expression from a specific layer, although there will be differing degrees of overlap from different layers (false positives). Fortunately, we have approximate quantifications for this from the ten-fold cross-validation. So if x > *precision*, randomly sample with replacement a gene from one of the predicted sets of layer-enriched genes (or, for conditional databases, from the

subset of genes having associated terms) with probabilities as calculated from an approximate solution to **Supplementary Equation 1**.

2. Repeat step 1 until the total number of genes in the simulated set is equal to the total number of predicted genes enriched in that layer (or the number of genes with associated terms for conditional databases).

3. For each term in the database, count the number of genes (allowing repeats) with that term. Save this and go back to step 1 until there are 200,000 resamples.

$$\begin{bmatrix} \Pr ecision_{2/3} & FP_{2/3}(4) & FP_{2/3}(5) & FP_{2/3}(6) & FP_{2/3}(6b) & FP_{2/3}(none) \\ FP_4(2/3) & \Pr ecision_4 & FP_4(5) & FP_4(6) & FP_4(6b) & FP_4(none) \\ FP_5(2/3) & FP_5(4) & \Pr ecision_5 & FP_5(6) & FP_5(6b) & FP_5(none) \\ FP_6(2/3) & FP_6(4) & FP_6(5) & \Pr ecision_6 & FP_6(6b) & FP_6(none) \\ FP_{6b}(2/3) & FP_{6b}(4) & FP_{6b}(5) & FP_{6b}(6) & \Pr ecision_{6b} & FP_{6b}(none) \\ FP_{none}(2/3) & FP_{none}(4) & FP_{none}(5) & FP_{none}(6) & FP_{none}(6b) & \Pr ecision_{none} \end{bmatrix} \begin{bmatrix} \Pr ob_k(2/3) \\ \Pr ob_k(4) \\ \Pr ob_k(5) \\ \Pr ob_k(6) \\ \Pr ob_k(6b) \\ \Pr ob_k(none) \end{bmatrix} = \begin{bmatrix} \Pr op_k(2/3) \\ \Pr op_k(4) \\ \Pr op_k(5) \\ \Pr op_k(6) \\ \Pr op_k(6b) \\ \Pr op_k(none) \end{bmatrix}$$

Supplementary Equation 1. Probabilities for simulating the false positives. $\text{Prob}_k(i)$ is the probability used to decide from which layer *i* a false positive for predicted layer *k* should be simulated (its value is 0 when i=k). $\text{FP}_k(i)$ is the probability that a gene predicted to be enriched in layer *i* was a false positive truly enriched in layer *k*. $\text{Prop}_k(i)$=$\text{FP}_k(i)$ for $k \neq i$ and needs to be calculated when i=k (using the fact that $\text{Prob}_k(i=k)$=0). $Precision_i$ is the precision of the classifier for layer *i* and is determined empirically from ten-fold cross-validation. FP_k(i) is determined empirically using the falsely called known genes. We do not use 'positives' by themselves because we wish to capture the effects of genes which are truly enriched in one layer but also happen to be truly enriched in a second layer, but not a third. We do not use higher-order (i.e. multi-layer) categories because, due to modest recall per layer, we were unable to reassemble those genes that have a complex multilayer pattern such as 2/3/5/6b with high accuracy. Instead, we use the same six categories used for classification: 2/3, 4, 5, 6, 6b, and no layer enrichment. Hence these values

and a normalization factor N are determined from **Supplementary Equation 2**. Since the exact solution results in negative sampling probabilities, we instead numerically minimized an error function (the sum of the squares of the deviation from the proper $\text{Prop}_k(i)$'s) with an iterative dense random sampling of the search space that consistently converged on the same solution.

$$\text{Precision}_k + N\sum_j FP_k(j) = 1$$ **(Supplementary Equation 2)**

Hence, $\text{Precision}_k + \sum_j \text{Prob}_k(j) = 1$

False negatives (measured by recall) are expected to adversely impact the power of this test to detect functional differences.

For all genes, the starting matrix was:

$$\begin{bmatrix} \text{Precision}_{2/3} & FP_{2/3}(4) & FP_{2/3}(5) & FP_{2/3}(6) & FP_{2/3}(6b) & FP_{2/3}(none) \\ FP_4(2/3) & \text{Precision}_4 & FP_4(5) & FP_4(6) & FP_4(6b) & FP_4(none) \\ FP_5(2/3) & FP_5(4) & \text{Precision}_5 & FP_5(6) & FP_5(6b) & FP_5(none) \\ FP_6(2/3) & FP_6(4) & FP_6(5) & \text{Precision}_6 & FP_6(6b) & FP_6(none) \\ FP_{6b}(2/3) & FP_{6b}(4) & FP_{6b}(5) & FP_{6b}(6) & \text{Precision}_{6b} & FP_{6b}(none) \\ FP_{none}(2/3) & FP_{none}(4) & FP_{none}(5) & FP_{none}(6) & FP_{none}(6b) & \text{Precision}_{none} \end{bmatrix} = \begin{bmatrix} 0.4440 & 0.2178 & 0.0381 & 0.0381 & 0.0598 & 0.1466 \\ 0.0688 & 0.3515 & 0.0196 & 0.000 & 0.0028 & 0.0114 \\ 0.1374 & 0.2275 & 0.6148 & 0.0810 & 0.1310 & 0.1949 \\ 0.0104 & 0.0339 & 0.0173 & 0.3805 & 0.1082 & 0.0465 \\ 0.0271 & 0.0097 & 0.0242 & 0.1954 & 0.3906 & 0.1115 \\ 0.3124 & 0.1597 & 0.2860 & 0.3050 & 0.3075 & 0.4890 \end{bmatrix}$$

The following probability columns were subsequently used in the simulations:

$$\begin{bmatrix} \text{Prob}_{2/3}(background) \\ \text{Prob}_{2/3}(4) \\ \text{Prob}_{2/3}(5) \\ \text{Prob}_{2/3}(6) \\ \text{Prob}_{2/3}(6b) \\ \text{Prob}_{2/3}(none) \end{bmatrix} = \begin{bmatrix} 0.4440 \\ 0.1192 \\ 0.0646 \\ 0.0013 \\ 0.0000 \\ 0.3708 \end{bmatrix}, \begin{bmatrix} \text{Prob}_4(2/3) \\ \text{Prob}_4(background) \\ \text{Prob}_4(5) \\ \text{Prob}_4(6) \\ \text{Prob}_4(6b) \\ \text{Prob}_4(none) \end{bmatrix} = \begin{bmatrix} 0.3203 \\ 0.3515 \\ 0.3130 \\ 0.0001 \\ 0.0013 \\ 0.0138 \end{bmatrix}, \begin{bmatrix} \text{Prob}_5(2/3) \\ \text{Prob}_5(4) \\ \text{Prob}_5(background) \\ \text{Prob}_5(6) \\ \text{Prob}_5(6b) \\ \text{Prob}_5(none) \end{bmatrix} = \begin{bmatrix} 0.0014 \\ 0.0438 \\ 0.6148 \\ 0.0020 \\ 0.0029 \\ 0.3350 \end{bmatrix},$$

$$\begin{bmatrix} \text{Prob}_6(2/3) \\ \text{Prob}_6(4) \\ \text{Prob}_6(5) \\ \text{Prob}_6(background) \\ \text{Prob}_6(6b) \\ \text{Prob}_6(none) \end{bmatrix} = \begin{bmatrix} 0.0008 \\ 0.0004 \\ 0.0060 \\ 0.3805 \\ 0.2593 \\ 0.3529 \end{bmatrix} \begin{bmatrix} \text{Prob}_{6b}(2/3) \\ \text{Prob}_{6b}(4) \\ \text{Prob}_{6b}(5) \\ \text{Prob}_{6b}(6) \\ \text{Prob}_{6b}(background) \\ \text{Prob}_{6b}(none) \end{bmatrix} = \begin{bmatrix} 0.0008 \\ 0.0121 \\ 0.0048 \\ 0.1277 \\ 0.3906 \\ 0.4640 \end{bmatrix}, \begin{bmatrix} \text{Prob}_{none}(2/3) \\ \text{Prob}_{none}(4) \\ \text{Prob}_{none}(5) \\ \text{Prob}_{none}(6) \\ \text{Prob}_{none}(6b) \\ \text{Prob}_{none}(background) \end{bmatrix} = \begin{bmatrix} 0.1233 \\ 0.0008 \\ 0.2601 \\ 0.0004 \\ 0.1257 \\ 0.4890 \end{bmatrix}$$

Replications in dorsal and lateral cortex

Laminar samples were dissected from dorsal cortex (overlapping S1 and some motor cortex) and, separately, lateral cortex (partially overlapping S2 and insular cortex) from mice matched in number, strain, sex, litter distribution, and age to the original set of mice. These additional eight adult male mice (56 days old; C57BL/6J strain) were also killed by cervical dislocation according to approved schedule one UK Home Office guidelines (Scientific Procedures Act, 1986). These were treated exactly as described above, and as described in the Experimental Procedures, with the following exceptions: sample B was not split and RNA underwent two rounds of poly(A) selection. Lanes with aberrant GC content were discarded and all reads were trimmed to correspond to two ends of 50 base pairs each. A read fragment was discarded if any of the following conditions was met: either read failed Illumina's chastity filter, either read had eight or more bases (corresponding to tophat's default minimum anchor length) with a Phred-scaled quality score less than three, either read mapped (bowtie v0.12.7 allowing up to two mismatches) to spliced or unspliced rRNAs, tRNAs or mitochondrial rRNAs as defined by Ensembl (release 62). Fragment internal insert size and standard deviation were estimated as described above. Fragments were subsequently mapped on a lane-by-lane basis with tophat v1.2.0 (Trapnell et al., 2009) enabling microexon search, coverage search, butterfly search, and providing gene models corresponding to Ensembl (release 62). All splice sites found in any lane (from original or replicate dorsal cortex samples, or lateral cortex samples) then combined for a final round of tophat mapping in which new splice site detection was disabled but all previously detected splice sites were provided. All lanes of the same libraries were merged and expression levels of all Ensembl genes (release 62) were quantified with cufflinks v0.9.3 (Trapnell et al., 2010), masking rRNA, tRNA and all mitochondrial transcripts from the denominator

for the FPKM calculation, and enabling the bias correction module (Roberts et al., 2011) and quartile normalization.

Once quantified, genes were filtered and then several characteristics were provided to a naïve Bayes classifier. First, genes were required to have a minimum FPKM of at least 1 in at least one replication dorsal cortex sample. Second, at least one relative error of the FPKM estimate (as provided by cufflinks) was required to be below 50%. Finally, the gene's quantification had to be marked as 'OK' by cufflinks in all samples. The following variables were then provided to the naïve Bayes classifier for each gene: average FPKM, % GC in the gene locus, enrichment status (if any) according to the Allen Mouse Brain Atlas manual curations, and, for every sample, the relative proportion of total expression in that sample and the relative error on that expression level. (Genes with no overlapping reads in a sample were defined to have a relative error of 10,000%, above any empirically observed relative errors for genes overlapping at least one read, in that sample to reflect to the classifier the high degree of uncertainty for such genes.) Classifiers were then trained, in turn, on these variables and metrics were reported as described above.

All classifiers were trained and assessed using data curated from S1 since expression patterns are known to be largely similar between dorsal and lateral cortex (Hawrylycz et al., 2010) and since naïve Bayes classifiers are robust to noisy data. Given that noisy test data will artificially depress the classifier's generalization measures, the fact that the AUCs for these classifiers, given in Table 2, are similar to the AUCs in dorsal cortex confirms again these assumptions were good.

Functional enrichments, as reflected in Figure 4B/C, were confirmed by performing Fisher's exact tests (one-tailed, in which the alternative hypothesis is that the gene set predicted to be enriched has more genes with that functional annotation

than does the gene set predicted not to be enriched). Functional differences between patterned and unpatterned genes, as reflected in Figure 4A, were confirmed by performing two-tailed Fisher's exact tests. These were appropriately tested as 'conditional' or 'nonconditional' databases, as described above. For a term to be tested, at least two genes were required to have the annotation and there must have been sufficient power to hypothetically achieve a *p*-value of at most 0.05. In both cases where the numbers were too large for R's Fisher's exact test function, the Chi-squared function was used instead.

Interestingly, using data from all samples from the S1 and dorsal cortex dissections simultaneously did not improve classifier performance. This suggests the reported generalized classifier performance may be limited by (1) artifacts, noise or mistakes in the *in situ* hybridization curations (for examples of these see Table S3), (2) fundamental differences in what is measured by *in situ* hybridization (analog expression within individual cells) and by RNA-seq (digital expression averaged across the sample), and/or (3) systematic biases in both sets of RNA-seq data or informatic pipelines. The first of these would cause the predicted probabilities to be overly conservative, and the effects of the second and third of these are already accounted for in the calibrated probabilities.

LincRNA substitution rates

We used mouse-human blastZ genome alignments (Schwartz et al., 2003), available from the UCSC Genome Browser Database (Rhead et al., 2010) to identify and extract the putative orthologous sequence in human (hg19) for all lincRNA loci and transposable element-derived Ancestral Repeats (AR) (Marques and Ponting, 2009). We considered only mouse-human alignments longer than 100 bp (907 lincRNA), to ensure the accuracy of the rate estimates. We estimated nucleotide

substitution rates (d_{loci} and d_{AR}) between orthologous mouse-human aligned sequences using baseml, from the PAML package (Yang, 1997), with the REV substitution model. To obtain normalized rates ($d_{\text{loci}}/d_{\text{AR}}$) we estimated the local neutral rates by concatenating all local ARs' mouse-human alignments from a matched G+C content class (Marques and Ponting, 2009). We then randomly sampled single columns from these alignments to obtain putatively neutral sequence with the exact length and nucleotide content as the sequence of interest and estimated the substitution rate. We used the median value from 1000 iterations to normalize d_{loci} and calculate ($d_{\text{loci}}/d_{\text{AR}}$).

LincRNA genome-wide association

To test the lincRNA-loci genome-wide association, we used a previously reported randomization procedure that accounts for G+C content and chromosome-specific biases (Marques and Ponting, 2009). We compared the observed density of cortical lincRNA transcription across 3 different types of annotations: *i*) DNAse I and histone methylation marks in neuronal precursor cells (Meissner et al., 2008; Mikkelsen et al., 2007); *ii*) RNA secondary structure predictions (Pedersen et al., 2006) and *iii*) patterned protein-coding gene territories against what would be expected based on the intergenic distribution of these annotations in the mouse genome. To define patterned protein-coding gene territories we first divided the mouse genome (mm9) into protein-coding gene (Ensembl build 59) territories by determining the mid-distance, *i*, between each known mouse protein-coding gene and its closest upstream and downstream protein-coding neighbour *i-1* and *i+1* (Ponjavic et al., 2009). A gene's territory is defined as the interval delimited by genomic coordinates *i-1* to *i+1*. Protein-coding genes associated with one or more patterned cortical transcript were annotated as patterned.

Correlation of expression with neighboring protein-coding genes

We identified the closest upstream and downstream cortical protein-coding transcript for each cortical lincRNAs and estimated the correlation (Spearman's rank correlation) of expression (FPKM) across samples between a lincRNA and its protein-coding neighbors using R. We considered significant correlations with an associated p-value < 0.025 (see also website).

***In situ* hybridization probes for Figure S7**

Fragments of each lincRNA target were amplified by RT-PCR from mouse whole brain cDNA or by PCR from genomic DNA and cloned into pCR4-TOPO (Invitrogen). Regions were selected to avoid repeat elements or possible sequence homology to other transcripts: nucleotides 70-490 of *EF177380* (*Malat1*), 508-914 of *AK159400* (*Neat1*) and 290-689 of *AK140632*. P56 male C57BL/6 mouse brains were frozen in OCT (VWR) on dry ice, and 10 μm cryosections were cut and mounted on positively charged slides. Sections were hybridized to anti-sense dioxygenin-labeled riboprobes as previously described (Isaacs et al., 2003). Slides were exposed for 16 hours with the exception of those using the *Malat1* riboprobe, which were hybridized for 2 hours.

Supplemental References

Bulfone, A., Smiga, S.M., Shimamura, K., Peterson, A., Puelles, L., and Rubenstein, J.L. (1995). T-brain-1: a homolog of Brachyury whose expression defines molecularly distinct domains within the cerebral cortex. Neuron *15*, 63-78.

Cleveland, W.S., and Devlin, S.J. (1988). Locally Weighted Regression: An Approach to Regression Analysis by Local Fitting. Journal of the American Statistical Association *83*, 596-610.

Croft, D., O'Kelly, G., Wu, G., Haw, R., Gillespie, M., Matthews, L., Caudy, M., Garapati, P., Gopinath, G., Jassal, B., *et al.* (2011). Reactome: a database of reactions, pathways and biological processes. Nucleic Acids Res *39*, D691-697.

Flicek, P., Amode, M.R., Barrell, D., Beal, K., Brent, S., Chen, Y., Clapham, P., Coates, G., Fairley, S., Fitzgerald, S., *et al.* (2011). Ensembl 2011. Nucleic Acids Res *39*, D800-806.

Hasan, M.R., Jin, M., Matsushima, K., Miyamoto, S., Kotani, S., and Nakagawa, H. (2006). Differences in the regulation of microtubule stability by the pro-rich region variants of microtubule-associated protein 4. FEBS Lett *580*, 3505-3510.

Inoue, K., Terashima, T., Nishikawa, T., and Takumi, T. (2004). Fez1 is layer-specifically expressed in the adult mouse neocortex. Eur J Neurosci *20*, 2909-2916.

Kent, W.J., Zweig, A.S., Barber, G., Hinrichs, A.S., and Karolchik, D. (2010). BigWig and BigBed: enabling browsing of large distributed datasets. Bioinformatics *26*, 2204-2207.

Li, H., Handsaker, B., Wysoker, A., Fennell, T., Ruan, J., Homer, N., Marth, G., Abecasis, G., and Durbin, R. (2009). The Sequence Alignment/Map format and SAMtools. Bioinformatics *25*, 2078-2079.

Meissner, A., Mikkelsen, T.S., Gu, H., Wernig, M., Hanna, J., Sivachenko, A., Zhang, X., Bernstein, B.E., Nusbaum, C., Jaffe, D.B., *et al.* (2008). Genome-scale DNA methylation maps of pluripotent and differentiated cells. Nature *454*, 766-770.

Mikkelsen, T.S., Ku, M., Jaffe, D.B., Issac, B., Lieberman, E., Giannoukos, G., Alvarez, P., Brockman, W., Kim, T.K., Koche, R.P., *et al.* (2007). Genome-wide maps of chromatin state in pluripotent and lineage-committed cells. Nature *448*, 553-560.

Nieto, M., Monuki, E.S., Tang, H., Imitola, J., Haubst, N., Khoury, S.J., Cunningham, J., Gotz, M., and Walsh, C.A. (2004). Expression of Cux-1 and Cux-2 in the subventricular zone and upper layers II-IV of the cerebral cortex. J Comp Neurol *479*, 168-180.

Ouimet, C.C., Miller, P.E., Hemmings, H.C., Jr., Walaas, S.I., and Greengard, P. (1984). DARPP-32, a dopamine- and adenosine 3':5'-monophosphate-regulated phosphoprotein enriched in dopamine-innervated brain regions. III. Immunocytochemical localization. J Neurosci *4*, 111-124.

Pavlidis, P., and Noble, W.S. (2003). Matrix2png: a utility for visualizing matrix data. Bioinformatics *19*, 295-296.

Roberts, A., Trapnell, C., Donaghey, J., Rinn, J.L., and Pachter, L. (2011). Improving RNA-Seq expression estimates by correcting for fragment bias. Genome Biol *12*, R22.

Schwartz, S., Kent, W.J., Smit, A., Zhang, Z., Baertsch, R., Hardison, R.C., Haussler, D., and Miller, W. (2003). Human-mouse alignments with BLASTZ. Genome Res *13*, 103-107.

Smedley, D., Haider, S., Ballester, B., Holland, R., London, D., Thorisson, G., and Kasprzyk, A. (2009). BioMart--biological queries made easy. BMC Genomics *10*, 22.
Tokuraku, K., Noguchi, T.Q., Nishie, M., Matsushima, K., and Kotani, S. (2007). An isoform of microtubule-associated protein 4 inhibits kinesin-driven microtubule gliding. J Biochem *141*, 585-591.
Watakabe, A., Ohsawa, S., Hashikawa, T., and Yamamori, T. (2006). Binding and complementary expression patterns of semaphorin 3E and plexin D1 in the mature neocortices of mice and monkeys. J Comp Neurol *499*, 258-273.
Xiong, H., Kovacs, I., and Zhang, Z. (2004). Differential distribution of KChIPs mRNAs in adult mouse brain. Brain Res Mol Brain Res *128*, 103-111.
Yang, Z. (1997). PAML: a program package for phylogenetic analysis by maximum likelihood. Comput Appl Biosci *13*, 555-556.
